# Pathogenic implications for autoimmune mechanisms derived by comparative eQTL analysis of CD4^+^ versus CD8^+^ T cells

**DOI:** 10.1371/journal.pgen.1006643

**Published:** 2017-03-01

**Authors:** Silva Kasela, Kai Kisand, Liina Tserel, Epp Kaleviste, Anu Remm, Krista Fischer, Tõnu Esko, Harm-Jan Westra, Benjamin P. Fairfax, Seiko Makino, Julian C. Knight, Lude Franke, Andres Metspalu, Pärt Peterson, Lili Milani

**Affiliations:** 1 Estonian Genome Center, University of Tartu, Tartu, Estonia; 2 Institute of Molecular and Cell Biology, University of Tartu, Tartu, Estonia; 3 Institute of Biomedicine and Translational Medicine, University of Tartu, Tartu, Estonia; 4 Divisions of Genetics and Rheumatology, Department of Medicine, Brigham and Women’s Hospital and Harvard Medical School, Boston, MA, United States of America; 5 Partners Center for Personalized Genetic Medicine, Boston, MA, United States of America; 6 Program in Medical and Population Genetics, Broad Institute of MIT and Harvard, Cambridge, MA, United States of America; 7 Wellcome Trust Centre for Human Genetics, University of Oxford, Oxford, United Kingdom; 8 Department of Genetics, University Medical Center Groningen, University of Groningen, Groningen, The Netherlands; New York Genome Center & Columbia University, UNITED STATES

## Abstract

Inappropriate activation or inadequate regulation of CD4^+^ and CD8^+^ T cells may contribute to the initiation and progression of multiple autoimmune and inflammatory diseases. Studies on disease-associated genetic polymorphisms have highlighted the importance of biological context for many regulatory variants, which is particularly relevant in understanding the genetic regulation of the immune system and its cellular phenotypes. Here we show cell type-specific regulation of transcript levels of genes associated with several autoimmune diseases in CD4^+^ and CD8^+^ T cells including a *trans*-acting regulatory locus at chr12q13.2 containing the rs1131017 SNP in the *RPS26* gene. Most remarkably, we identify a common missense variant in *IL27*, associated with type 1 diabetes that results in decreased functional activity of the protein and reduced expression levels of downstream *IRF1* and *STAT1* in CD4^+^ T cells only. Altogether, our results indicate that eQTL mapping in purified T cells provides novel functional insights into polymorphisms and pathways associated with autoimmune diseases.

## Author summary

Variation in regulatory regions as well as coding regions of the genome can affect the expression of genes. Many of these variants have been associated with different diseases and other traits, but the underlying biological pathways are often left unknown. Analysing the effect of single nucleotide polymorphisms (SNPs) on gene expression levels, referred to as expression quantitative trait loci (eQTL), in specific cell types can be used to gain insight into specific mechanisms of disease. By analyzing eQTLs in CD4^+^ and CD8^+^ T cells, essential elements of adaptive immune response, we identified both *cis*- and *trans*-acting SNPs in genes associated with several autoimmune diseases.

## Introduction

T cells are essential elements of the adaptive immune response [1]. CD4^+^ T cells, together with an appropriate cytokine environment, are required for the activation and differentiation of CD8^+^ T cells that mediate defense and pathogen clearance during various infections [2]. The role of CD4^+^ T cells is also necessary for B cells and macrophages to execute their protective functions.

T cells are activated and start to differentiate in response to complex stimuli involving cytokines, membrane receptors and transcription factors. Signaling through T cell receptor and costimulatory molecules induces their rapid proliferation and clonal expansion, and differentiation into effector and memory T cell subtypes [3]. Faulty activation or inadequate regulation of CD4^+^ and CD8^+^ T cells may contribute to the initiation and progression of multiple autoimmune diseases, including type 1 diabetes (T1D), rheumatoid arthritis (RA), autoimmune thyroiditis, systemic lupus erythematosus (SLE), multiple sclerosis, psoriasis, inflammatory bowel disease, as well as allergy and asthma [4,5]. In addition, a distinct lineage of CD4^+^ T cells, regulatory T cells, have a central role in the control of the pathogenesis of autoimmune and inflammatory diseases [6].

Genome-wide association studies (GWAS) have identified thousands of single nucleotide polymorphisms (SNPs) associated with various immune related diseases [7–12]. Studies of the consequences of the risk alleles in biologically relevant contexts for different diseases have stressed the importance of the tissue and cell type-specificity of the regulatory variants [13–15]. We have recently shown that by mapping eQTLs in a sufficiently large sample set, it is possible to identify cell type-specific effects in whole blood, but the challenge to distinguish the cells responsible for the associations remains [16,17]. The power of studying expression-associated genetic variants in purified cells types has now been illustrated for B cells, monocytes, neutrophils, T regulatory cells and CD4^+^ T cells [18–21], which allowed the identification of functional roles for several polymorphisms at autoimmune and even neurodegenerative disease loci. Meanwhile, some particularly interesting *cis*- and *trans*-eQTLs identified in whole blood could not be attributed to these cell types, and require an expanded survey of cells involved in immune response.

To this end, we purified CD4^+^ and CD8^+^ T cells from the peripheral blood of 313 healthy individuals for genome-wide mapping of genetic variation affecting the expression of genes involved in immune response. Our analysis characterizes both the extent of genetic control of gene expression in T cells and its cellular specificity, as well as variation in isoform levels of genes and their association with epigenetic changes. We show that integrating knowledge from GWAS with eQTLs enables us to clarify the functional consequences of disease-associated variants and assess the enrichment of autoimmune response. Finally, we highlight a common T1D-associated missense variant in *IL27* affecting the STAT1 and IRF1 pathway in CD4^+^ T cells. Our analysis provides insights into the basic processes of the regulation of gene expression in T cells and advances our understanding about the pathways involved with disease susceptibility in the adaptive immune system.

## Results

### Genetic control of gene expression in T cells

We purified CD4^+^ and CD8^+^ T cells from peripheral blood mononuclear cells (PBMCs) of 313 healthy European individuals from the Estonian Biobank [22]. The purified cells were subjected to genome-wide gene expression analysis, genotyping and imputation using the 1000 Genomes reference panel. After stringent quality control and filtering, close to 6 million SNPs, and expression data from 38,839 probes within 23,704 genes were included in the analysis. DNA methylation of 450,000 CpG sites was determined in the same purified cells of 100 of the individuals [23] to add explanations to observed differential expression between cell types.

**Fig 1:**
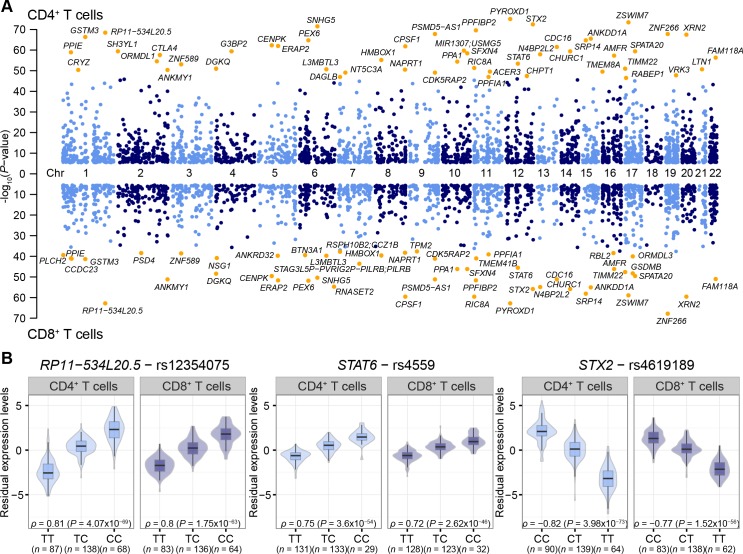
Summary of *cis*-eQTL analysis in CD4^+^ and CD8^+^ T cells. **(A)** Manhattan plots of the significant *cis*-eQTLs per gene in CD4^+^ (top) and CD8^+^ T cells (bottom). The names of the top 50 genes per cell type are shown. (**B**) Examples of genes with *cis*-eQTLs: *RP11−534L20*.*5* is located downstream of the *IKBKE* gene which has an essential role in regulating inflammatory responses to viral infections; *STAT6* is involved in T cell differentiation; and *STX2* is involved in intracellular transport of vesicles. The allelic effect of the SNPs on gene expression levels are shown by boxplots within violin plots. Violin plot shows the density plot of the data on each side, the lower and upper border of the box correspond to the first and third quartiles, respectively, the central line depicts the median, and whiskers extends from the borders to ±1.5xIQR, where IQR stands for inter-quantile range, the distance between the first and third quantiles.

To characterize the extent of genetic control of gene expression in T cells and its cellular specificity, we first tested the association between SNPs and gene expression within 1Mb intervals, referred to as *cis*-eQTLs. In total, we identified *cis* regulatory SNPs for 2,605 genes in CD4^+^ T cells and 2,056 genes in CD8^+^ T cells at probe-level false discovery rate (FDR) < 0.05 (**Fig 1A**, **S1 Table**), with an overlap of 1,637 genes and estimated replication rate π_1_ = 0.99. The high replication rate reflects the similarity of the two cell types compared, and also highlights the limitations of arbitrary cut-off levels for significance. Moreover, about half of the significant eQTLs detected in a large meta-analysis of peripheral blood from 5000 individuals [16] could be replicated in the CD4^+^ and CD8^+^ T cells (π_1_ = 0.51 and 0.45, respectively, **S1 Fig**), indicating a high level of T cell-specific eQTL effects [24,25]. As examples of *cis*-eQTLs in CD4^+^ and CD8^+^ T cells, we observed evidence of genetic regulation of long coding RNA *RP11−534L20*.*5* located 7 kb downstream of the *IKBKE* gene which has an essential role in regulating inflammatory responses to viral infections; *STAT6*, a transcriptional activator involved in T cell differentiation, and *STX2* (syntaxin 2), involved in intracellular transport of vesicles (**Fig 1B**).

### Cell type-specificity of eQTLs

We next used a Bayesian model averaging and hierarchical modelling [26] that combines information across genes to jointly call eQTLs in one framework to assess the specificity and proportion of shared eQTLs in CD4^+^ and CD8^+^ T cells. Of 3871 genes associated with eQTLs, all showed very strong posterior probability to be shared between CD4^+^ and CD8^+^ T cells (**S2 Table**).

One of the strengths of the multi-tissue joint *cis*-eQTL analysis is its capability to account for incomplete power [26,27]. As our sample size for both of the cell types was similar, we noted the advantage of Bayesian methods in particular for genes with eQTLs that have modest effects. Moreover, for eQTLs where the absolute effect size was higher in one cell type, there was also a tendency for higher expression levels in that gene in the given cell type (chi-square test *P*-value < 2x10^-16^, **S2 Fig**). For a standard tissue-by-tissue analysis, higher effect sizes and small standard deviations lead to more statistical evidence against the null, thus resulting in lower *P*-values.

**Fig 2:**
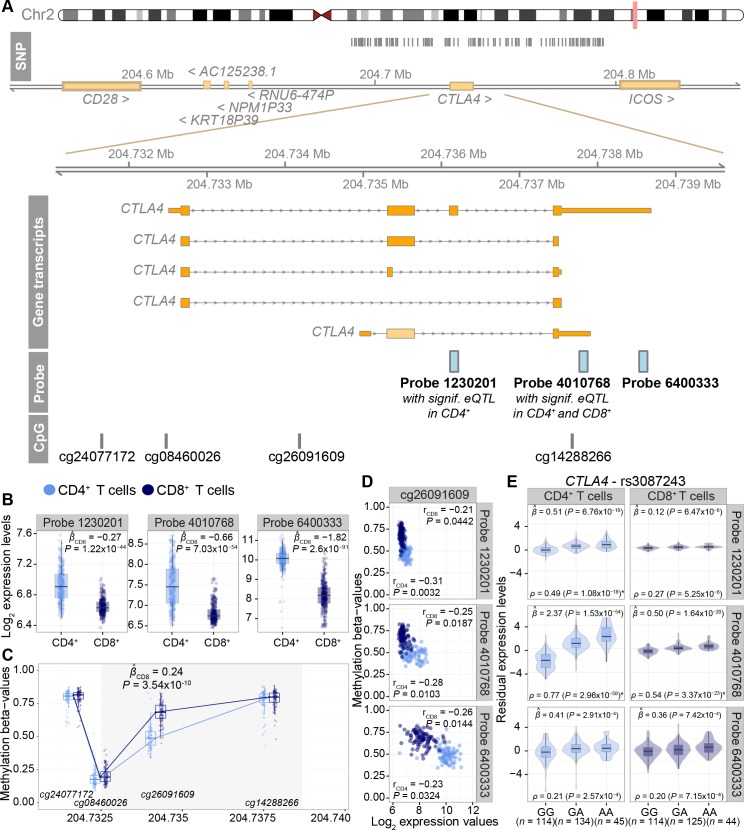
Probe-level differences between CD4^+^ and CD8^+^ T cells in the *CTLA4* gene. (**A**) Overview of the *CTLA4* gene locus. On the top panel the SNPs associating with different isoform levels between the two cell types in the *CTLA4* gene are depicted as grey bars, the gene transcripts are drawn, and the positions of the three gene expression probes and four CpG sites in the region are shown at the bottom. (**B**) The expression levels (*y*-axis, quantile normalized and log_2_ transformed) for each of the probes are shown by boxplots for CD4^+^ and CD8^+^ T cells. The mean of the differences in expression levels between CD4^+^ and CD8^+^ T cells are estimated by linear mixed models. (**C**) Methylation levels (*y*-axis) for each of the CpG sites along the genomic position (*x*-axis) are shown by boxplots for CD4^+^ and CD8^+^ T cells. Medians of the methylation values per CpG sites are connected by a line in both cell types. The mean of the differences in methylation levels between CD4^+^ and CD8^+^ T cells are estimated by linear mixed models. Only estimates with *P*-value < 0.05 are marked on the figure. (**D**) Correlation between methylation and gene expression levels are shown on the scatter plot between the differentially methylated CpG site cg26091609 for CD4^+^ and CD8^+^ T cells (*y*-axis) and three expression probes (*x*-axis). (**E**) The effect of rs3087243 on the expression levels (*y*-axis, residual gene expression levels) of different probes in the *CTLA4* gene are shown by boxplots within violin plots depicted as in **Fig 1.** Effect sizes and corresponding *P*-values are reported for the multi-tissue and tissue-by-tissue analysis above and below of the violin plots, respectively. Significant eQTLs in the tissue-by-tissue analysis are indicated with a star (*).

The effect of the magnitude of the expression levels is exemplified by the effect sizes and *P*-values of the probes in the inhibitory immune checkpoint gene, *CTLA4*, known to be associated with T1D and other endocrine autoimmune diseases [28,29]. *CTLA4* is covered by three probes all showing consistent eQTL effects shared by both cell types in the multi-tissue analysis (**Fig 2A)**. In the tissue-by-tissue analysis, expression levels of *CTLA4* were associated with 122 SNPs (**S3A Fig**), including T1D and RA-associated variants rs231775 (+49A/G) [28], rs3087243 (CT60A/G) [28,30], rs231806 (MH30C/G) [28], and rs231735 [8] (pair-wise r^2^ between the SNPs ranging from 0.51 to 0.90, and strong linkage disequilibrium (LD, r^2^ > 0.8) between MH30 and CT60, **S3C Fig**). The CT60 polymorphism is also in strong LD with length variation of the (AT)n repeat in the 3’UTR of *CTLA4* with long variants of the repeat associating with lower *CTLA4* mRNA expression in autoimmune T cell clones [31]. Our results extend this finding to the polyclonal population of CD4^+^ and CD8^+^ T cells, where the disease risk alleles were associated with lower expression levels of *CTLA4* (**S3B Fig**). Genetic control of the expression of the alternatively spliced third exon, that is omitted from the secretory variant of *CTLA4* [28], was significantly detectable in CD4^+^ T cells only, whereas genetic association with the expression of the fourth exon was significant in both cell types (**Fig 2B and 2E**). Further investigation of the *CTLA4* gene locus revealed differential methylation between CD4^+^ and CD8^+^ T cells (**Fig 2C**). Specifically, methylation of a CpG site near the second exon of *CTLA4* (cg26091609) was inversely correlated with the expression levels of the three different probes in the gene (**Fig 2D**), possibly explaining the expression differences in CD4^+^ and CD8^+^ T cells, and thus failing to detect significant eQTL effects in the tissue-by-tissue analysis.

Interestingly, divergent DNA methylation between CD4^+^ and CD8^+^ T cells was similarly detectable for an additional 73 out of 187 genes covered by more than one probe, and with seemingly isoform-specific eQTL effects (**S3 Table**). Among the 74 genes, multi-tissue analysis resulted in the identification of eQTL effects for two or more probes in 66 genes which were shared between the cell types. These observations suggest that the differential splicing effects, plausibly regulated by epigenetic DNA methylation, influence the gene expression levels, and thus the power to significantly detect eQTLs in the tissue-by-tissue analysis.

### T cell specific trans-acting regulatory locus

**Fig 3:**
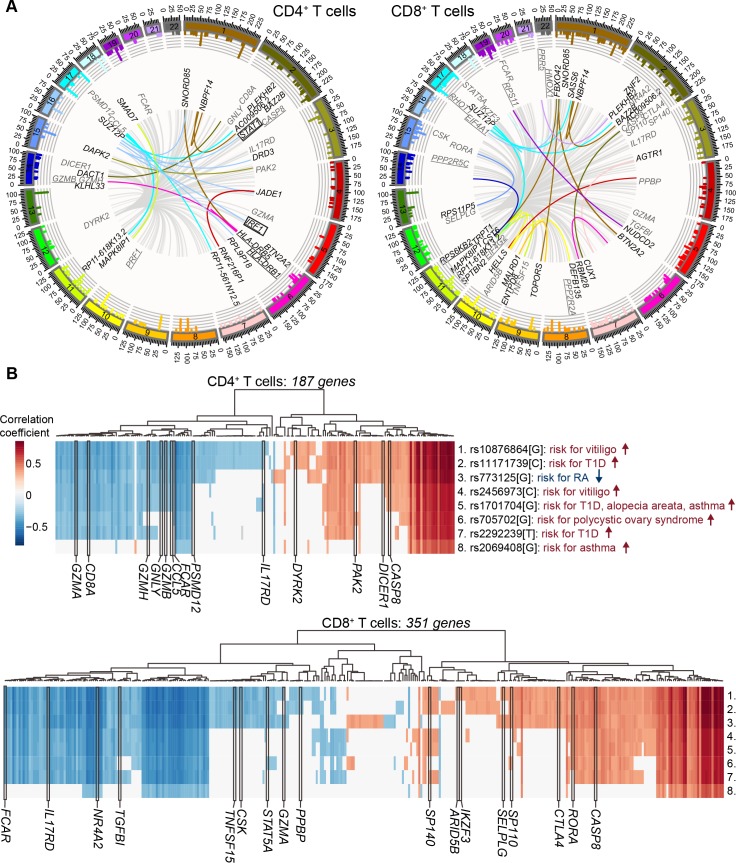
T*rans*-associations in CD4^+^ and CD8^+^ T cells. (**A**) The outermost rim of the circos plot shows the histogram of the –log_10_(*P*-value) of the associations between known genes and SNPs. Per every significant known gene only the highest –log_10_(*P*-value) is depicted. The innermost network represents the *trans*-associations between the SNPs and the most significant gene expression probes per gene. The lines are colored by the chromosome of the given SNP, except for the locus on chr12q13.2 with over 100 *trans*-associations that are colored in grey. An arbitrary selection of genes is depicted. All genes not affected by chr12q13.2 SNPs are colored in black and a set of genes affected by chr12q13.2 *trans*-acting regulatory SNPs are selected based on their known importance in immune system related processes and are colored in grey. The underlined genes are involved in T1D and mTOR signaling in CD4^+^ T cells and CD8^+^ T cells, respectively. Two boxed genes among the genes with *trans*-eQTLs in CD4^+^ T cells are associated with the T1D susceptibility variant rs4788084 close to the *IL27* gene. (**B**) Heatmap of the correlation coefficients between chr12q13.2 *trans*-acting regulatory SNP allele dosages and gene expression levels in CD4^+^ and CD8^+^ T cells are shown. The most significant gene expression probe per gene is chosen. An arbitrary selection of genes based on their importance in immune system related processes are marked with black borders and gene symbol. The depicted SNPs are linked with their role in disease susceptibility based on GWAS studies [30,33–40] and the numbers indicated in the lower panel refer to the same SNPs listed in the top panel.

For the analysis of SNPs affecting the expression of distal genes (>5 Mb apart), referred to as *trans*-eQTLs, we selected all 4,638 genome-wide significant (*P*-value < 5x10^-8^) SNPs from the GWAS catalog [32] (accessed 24/03/2015). After correcting gene expression levels for *cis*-eQTL effects, we identified 36 and 40 GWAS SNPs associated with the expression levels of 209 and 378 distant genes in CD4^+^ and CD8^+^ T cells, respectively (overlap of 21 SNPs and 133 genes; **Fig 3A**, **S4 Table**). The functions of the genes associated with the *trans*-acting GWAS SNPs highlighted their role in T1D (Ingenuity pathway analysis, *P* = 4.39x10^-5^, **Fig 3A**) and mTOR signaling (*P* = 3.84x10^-3^, **Fig 3A**) in CD4^+^ and CD8^+^ T cells, respectively.

Strikingly, we observed a *trans*-acting regulatory locus at chr12q13.2 in CD4^+^ and CD8^+^ T cells with broad-range impact on hundreds of genes. Five SNPs in that region were previously implicated in B cell-specific trans-associations by Fairfax *et al*. [18]. In T cells, we identified the lead SNP in this region as rs1131017 located in the 5´UTR of the ribosomal small subunit protein *RPS26* gene (**S5 Table**). The rs1131017 SNP is in LD with eight GWAS SNPs implicated in T1D [34,36,40], vitiligo [33,35] and other autoimmune and inflammatory diseases [30,37–39]. The pair-wise r^2^ values of the nine SNPs are shown in **S4B Fig** and their eQTL effects in **S4C Fig**, illustrating the strong LD (r^2^ > 0.8) between the top *trans*-eQTL SNP rs1131017 and several of the GWAS SNPs. In addition to *RPS26*, the *trans*-acting region contains the *CDK2*, *RAB5B*, *SUOX*, *IKZF4* and *ERBB3* genes (**S4A Fig**) and associates with the expression levels of 187 and 351 genes in CD4^+^ and CD8^+^ T cells, respectively, with an overlap of 124 genes (**Fig 3B)**. Many of these genes are highly expressed and have specific roles in T cells such as *CTLA4*, *GZMA*, *GZMB*, *GZMH*, *GNLY* and *CD8A* (**Fig 3B**).

### Missense variant in *IL27* as a candidate disease variant for T1D revealing significant *trans*-eQTL effects in CD4^+^ T cells

**Fig 4:**
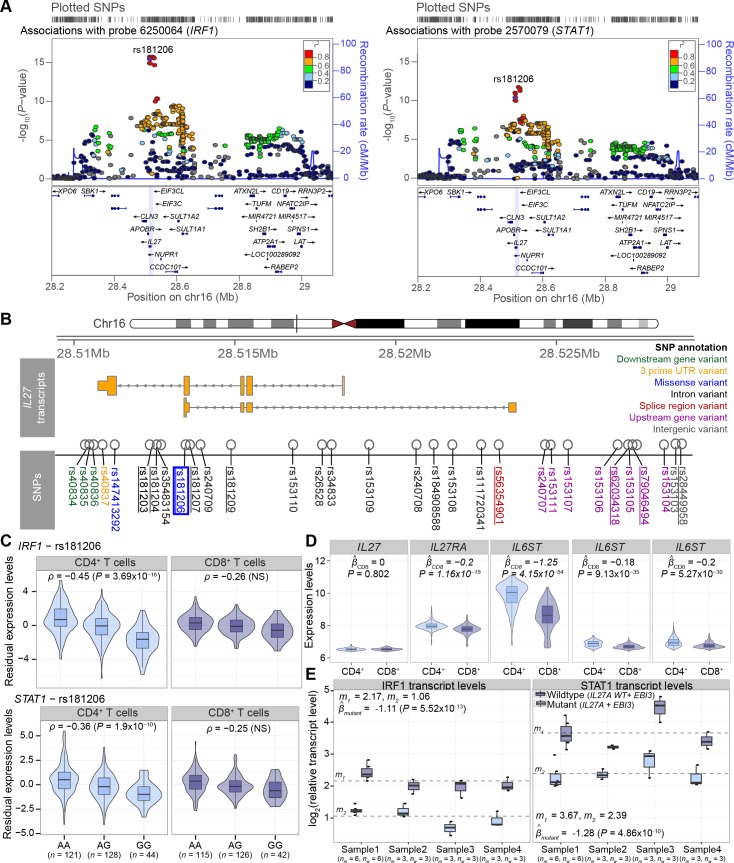
*Trans* associations with *IRF1* and *STAT1* gene expression levels in CD4^+^ T cells. (**A**) Regional association plots of the *IL27* region SNPs with association *P*-values for expression levels of *IRF1* (chr5) and for *STAT1* (chr2) in CD4^+^ T cells. The missense SNP rs181206 is used as the index SNP for showing LD between the SNPs. (**B**) Overview of the *IL27* transcripts and SNPs located in the *IL27* gene region. In addition to the missense SNP rs181206, other SNPs with the lowest association *P*-values in the upper LD cloud in panel (**A**) are intronic variants rs181203, rs181204, rs181207, rs181209, splice region variant rs56354901, upstream gene variants rs62034318, rs79046494, and intergenic variant rs28449958. (**C**) The effect of the missense SNP rs181206 on *IRF1* and *STAT1* gene expression levels are shown. *P*-values greater than 9.2x10^-8^ in CD8^+^ T cells resulted in FDR > 0.05 noted as NS (not significant). (**D**) Expression levels (*y*-axis, quantile normalized and log_2_-transformed) grouped by cell type (*x*-axis) are shown for the three genes: *IL27* (probe 6520523 in the last exon), *IL27RA* gene (probe 4250735 in 3' downstream sequence), and *IL6ST* gene (probe 4010100 at the end of the last exon, 3830048 at the beginning of the last exon, 4260333 in the middle of the gene, from left to right). Linear mixed effects model is used to estimate the mean of the differences in expression levels between CD4^+^ and CD8^+^ T cells. (**E**) The effect of the mutant and wild-type alleles of rs181206 on the expression levels of *IRF1* and *STAT1*. The log_2_ relative transcript levels (*y*-axis) are shown as a boxplot per allele and sample, with four samples in total. Every sample was run in multiple parallel reactions, indicated by *n*_*m*_ (number of mutant reactions) and *n*_*w*_ (number of wild-type reactions), Sample1 was used in two parallel sets. The mean expression in each class is shown by grey dashed lines, where *m*_*1*_ and *m*_*2*_ are indicating the mean among wild-type and mutant samples, respectively. The effect of the mutant SNP on transcript levels is evaluated by linear mixed effects models. Boxplots within violin plots are depicted as in **Fig 1**.

We also identified a *trans*-eQTL (GWAS SNP rs4788084[T]) on chr16p11.2 close to the *IL27* gene associated with lower expression of *IRF1* (*P =* 1.84x10^-9^) and *STAT1* (*P =* 2.91x10^-8^) in CD4^+^ T cells only. The rs4788084[T] is associated with lower expression of *STAT1* in peripheral blood [16] and reduced risk for T1D [7,11]. To further explore the effects of genetic variants in the *IL27* region, we mapped *trans*-eQTLs for all SNPs in the chromosome 16:28,2–29,1 Mb region (829 SNPs in total, **S6 Table, Fig 4A**).

This analysis revealed an even stronger *trans*-eQTL signal for a missense SNP rs181206[G] within the *IL27* gene (**Fig 4B**) and decreased expression levels of *IRF1*, *STAT1* and *REC8* (**Fig 4C**). The rs181206[G] allele, with a minor allele frequency of 29% in the 1000 Genomes phase 3 European population, causes the amino acid Leu119Pro change in the alpha-helical domain of IL-27 (**S5 Fig**). Strong LD (r^2^ ranges from 0.62 in the Estonian population to 0.84 in the other European populations, 1000 Genomes phase 3) between rs181206 and the GWAS SNP rs4788084 supports the protective role of the rs181206[G] allele for T1D among individuals of European ancestry. Of note, the effect of the missense SNP on *IRF1* and *STAT1* remains after regressing out the effect of the GWAS SNP, but not the other way around. Moreover, Bayesian test for colocalisation [41] adds strength to the hypothesis that T1D susceptibility and changes of expression in *IRF1* and *STAT1* are associated with the region, and share a single causal variant with the most posterior support for rs181206[G] (**S7 Table**).

Importantly, despite the relatively small sample size, our focus on T cell subtypes enabled us to identify this effect as specific to CD4^+^ T cells, as we did not detect significant *trans*-eQTL effects in this region in CD8^+^ T cells (**S6 Fig**). We also confirmed the absence of the signal in B cells and monocytes by re-analyzing the data from Fairfax *et al*. [18] (**S6 Fig**). Notably, the *trans*-eQTL locus had no effects on the expression level of *IL27* in *cis* (**S7 Fig**). In agreement with the cell type-specific effect, we found higher expression levels of the *IL27RA* and *IL6ST* (gp130) genes, which together act as a receptor for the IL-27 cytokine, in CD4^+^ cells in comparison to CD8^+^ T cells (**Fig 4D**).

Given the strong positive correlation between the expression levels of *IRF1* and *STAT1*, we used structural equation modeling to determine whether the *IL27* SNP rs181206 affects both genes independently or via each other. Overall, the best-fitting scenario suggested *IRF1* to mediate the SNP and *STAT1* relationship (model 1, **S8 Table**). The finding was supported by a simulation experiment (**S8 Fig**) and suggested a mechanism for the effect of IL-2*7* on *IRF1* and *STAT1* expression (**S9 Fig**).

### Functional effects of the *IL27* missense SNP suggest causal role in decreased expression of IRF1 and STAT1

In order to confirm the functional effect of the *IL27* rs181206[G] allele we investigated its effect on *IRF1* and *STAT1* expression in human PBMCs by additional experiments. IL-27 is produced by innate immune cells, and after forming a heterodimer with EBI3, it interacts with its receptor IL27RA and activates the STAT1/STAT3 pathway in T cells [42]. After binding to interferon stimulated response elements (ISRE), the STAT1/STAT3 pathway induces transcription of several interferon-induced genes, including IRF1 and STAT1 itself. We cloned cDNA variants of the IL-27 wild-type (Leu119) and missense (Pro119), as well as EBI3. After transfection into HEK293 cells, we combined the cell supernatants containing either IL-27 Leu119 or Pro119 protein with an equal amount of EBI3 protein and studied their effect on *IRF1* and *STAT1* expression by real-time PCR in human PBMCs from four healthy individuals.

As shown in **Fig 4E**, the missense SNP resulting in Pro119 in IL-27 induced lower *STAT1* and *IRF1* transcript levels compared to IL-27 Leu119. The comparison of the fixed effects of the rs181206[G] allele resulted in highly significant estimates: ${\hat{\beta }}_{mutant}$ = -1.11 (*P* = 5.52x10^-13^) for IRF1 and ${\hat{\beta }}_{mutant}$ = -1.28 (*P* = 4.86x10^-10^) for STAT1. This result supports our *trans*-eQTL analysis, suggesting that the Pro119 in the *IL27* gene is the causal SNP of these associations.

## Discussion

We here report eQTL mapping in purified CD4^+^ and CD8^+^ T cells and reveal multiple effects on regulation of genes associated with autoimmune diseases. The eQTL studies in cell subtypes have both demonstrated a high level of specificity as their effects vary across cell types, as well as remarkable sensitivity despite their several fold smaller sample sizes [24,25]. Indeed, we also observed many *cis*-eQTLs identified in CD4^+^ and CD8^+^ T cells that were shared according to the joint analysis, but were seemingly cell type-specific by the tissue-by-tissue analysis. The genes with such notable patterns include *CTLA4*, known for its inhibitory role in T cell mediated immune responses and association with many organ-specific autoimmune diseases [29]. The autoimmunity-susceptible SNPs in this region (rs231775, rs3087243, and rs231806) have been associated with various functional effects including *CTLA4* transcript levels, splicing, production of the soluble form of CTLA-4 and posttranslational modifications [28,43–45]. In addition, our results show differential DNA methylation in the *CTLA4* region to be associated with the potential isoform-specific differences in CD4^+^ and CD8^+^ T cells. This is likely due to negative correlation between DNA methylation and gene expression, which eventually leads to lower expression levels and moderate effect sizes. To confirm whether the observed effects are due to true differences in isoforms instead of the quality of the designed probes at capturing variation in gene expression levels, a follow-up study with RNA-seq data is needed.

The observed *trans*-acting regulatory region on chr12q13.2 in CD4^+^ and CD8^+^ T cells indicates the important role of genetic variants in that region affecting the expression levels of over a hundred genes across the genome. Interestingly, the region includes the *RPS26* gene, which may constitute a mechanism for the detected *trans*-eQTL effects. Our lead SNP rs1131017 is located in an oligopyrimidine tract of the 5´UTR of the *RPS26* mRNA and its T1D risk allele rs1131017[C] correlates positively with *RPS26* expression levels [46,47]. The oligopyrimidine tract controls the translation of many mammalian ribosomal protein genes [48], and the effect of the SNP on *RPS26* ribosomal distribution has been reported [49]. RPS26 is a main component of the ribosomal region involved in the recruitment of cellular mRNA during translational initiation and in maintenance of the path of mRNA molecules to the ribosomal exit site [50]. Hence, it is conceivable that changed RPS26 protein levels may affect the stability or translational efficiency of a large number of cytosolic mRNAs. Nevertheless, the exact functional role of rs1131017[C] in T1D remains to be identified, as well as the effect of other candidate genes in this region such as *IKZF4*, *ERBB3* [51] and *CDK2* [52].

Strikingly, we identified a common missense variant in cytokine *IL27* as a significant *trans*-eQTL for *IRF1* and *STAT1* in CD4^+^ T cells. The effect of IL-27 in T cells is regarded as anti-inflammatory but it has also been shown as a growth and survival factor for T cells [42]. As a heterodimer with EBI3, IL-27 activates the STAT1/STAT3 pathway that induces the transcription of several interferon-induced genes, including *IRF1* and *STAT1* itself. Our model suggests that *IRF1* mediates the effect of the *IL27* SNP on expression levels of *STAT1*. Moreover, our functional studies with the mutated form of IL-27 confirmed its decreased capacity to activate the STAT1 pathway, and we showed that a potential causal variant (the missense variant rs181206) for T1D susceptibility and changes in *IRF1* and *STAT1* expression is shared. Furthermore, our findings are supported by studies of a T1D mouse model with high levels of IL-27 and delayed T1D onset after treatment with an IL-27 blocking antibody [53]. Altogether these results suggest that the rs181206[G] variant of the *IL27* gene confers protection against T1D through the inhibited expression of IRF1 and STAT1 in CD4^+^ T cells. Our results also suggest that IL-27 may promote autoimmunity toward pancreatic islets via the upregulation of the STAT1 pathway.

In conclusion, the analysis of genetic modulators of gene expression profiles as intermediate phenotypes between human traits and underlying genetic variation offers new instruments to refine our understanding of disease susceptibility. Moreover, eQTL studies in purified cell types instead of whole blood enable us to establish the specific mechanisms and pathways involved in diseases progression and create the basis for future explorations and drug interventions.

## Materials and methods

### Study design

The aims of this study were to perform *cis*- and *trans*-eQTL mapping in purified CD4^+^ and CD8^+^ T cells and evaluate their pathogenic implications for autoimmune mechanisms. Based on similar studies [18], we considered the sample size of 300 individuals to be sufficient to ensure statistical power to detect eQTL effects in purified cells. This study participants were healthy donors of the Estonian Genome Center of the University of Tartu [22]. In total, 313 subjects were selected for the study, with median age 54 (standard deviation 17.8), 154 females and 159 males. The study was approved by the Ethics Review Committee of Human Research of the University of Tartu, Estonia (permission no 206/T-4, date of issue 25.08.2011) and it was carried out in compliance with the Helsinki Declaration. A written informed consent to participate in the study was obtained from each individual prior to recruitment. All methods were carried out in accordance with approved guidelines.

DNA from the samples were genotyped using HumanOmniExpress BeadChips (Illumina), according to the manufacturer’s instructions. We imputed both datasets using the 1000 Genomes project reference by using IMPUTE v2 [54], resulting in 5,879,386 autosomal SNPs with a minor allele frequency (MAF) of > 0.05 for downstream analyses. CD4^+^ and CD8^+^ T cells were extracted from the peripheral blood mononuclear cells (PBMC) by consecutive positive separation using microbeads (CD4^+^ #130-045-101; CD8^+^ #130-045-201) and AutoMACS technology (Miltenyi Biotec) according to the manufacturer's protocol. Gene expression data was generated using HumanHT-12v4 BeadChips (Illumina), according to the standard protocol. Preprocessing and quality control of the data was done using R [55] and the Bioconductor packages *lumi* [56] and *arrayQualityMetrics* [57]. The number of samples retained for further analysis was 293 for CD4^+^ and 283 for CD8^+^ T cells, 303 unique individuals. The effect of the mutant and wild-type *IL27* was experimentally tested in HEK 293 cells. The procedures are described in details in the **S1 File.**

### *Cis*- and *trans*-eQTL mapping

The effects of SNPs on local (*cis*-eQTL) and distant (*trans*-eQTL) genes were determined via eQTL mapping as described in eQTL mapping analysis cookbook developed by the University Medical Center Groningen at the Genetics Department and the Genomics Coordination Center (https://github.com/molgenis/systemsgenetics/wiki/eQTL-mapping-analysis-cookbook) and previously in [16]. For *cis*-analysis the distance between the probe midpoint and SNP genomic location was up to 1 Mb and for *trans*-analysis the distance was more than 5 Mb or the probe and SNP were on different chromosomes. Only SNPs with a MAF > 0.05, call rate > 0.95 and a Hardy-Weinberg equilibrium *P*-value > 0.001 were included in the analyses. We used the Spearman correlation coefficient (Spearman’s rho) to detect associations between the coding allele of the SNP (directly genotyped or imputed allele dosages) and the variations in gene expression levels (residual gene expression levels obtained after corrections as described in “Gene expression quality control and normalization” in Supplementary Methods). To control for multiple testing, we applied more conservative probe-level false discovery rate (FDR) procedure. The eQTL mapping procedures are described in details in the **S1 File**.

### Proportion of true positives

To estimate the sharing of *cis*-eQTLs between the two cell types and peripheral blood we used the π_1_ statistic (Storey and Tibshirani *q*-value approach [58] implemented in the R *qvalue* package, default settings used). Based on the list of *P*-values, the overall proportion of true null hypotheses (π_0_) is estimated, i.e. those following the Uniform(0,1) distribution. An estimate of true alternative tests is π_1_ = 1 – π_0_, i.e. π_1_ is the proportion of true positives. The reported replication rate (proportion of sharing) between two groups (CD4^+^ or CD8^+^ T cells or pheripheral blood) is the π_1_ statistic estimated by taking the significant SNP-probe list from one group and using the corresponding *P*-value distribution in the other group.

### Multi-tissue joint discovery of eQTLs

We used Bayesian framework for multi-tissue joint eQTL analysis by Flutre *et al*. [26] in CD4^+^ and CD8^+^ T cells implemented in the eQtlBma program. The *cis* candidate region was defined as +/- 1 Mb from probe midpoint. As an input to the program, residual gene expression levels obtained after corrections as described above and the corresponding genotypes were used. Firstly, the raw Bayes factors for each configuration (eQTL active in CD4^+^, eQTL active in CD8^+^, eQTL active in both cell types) were computed assessing the support in the data for each probe-SNP pair being an eQTL. Secondly, information across all genes were combined by the hierarchical model with an EM algorithm to get maximum-likelihood estimates of the configuration probabilities. Thirdly, by Bayesian model averaging using the raw Bayes factors weighted by the estimated configuration probabilities, the posterior probabilities for the eQTL to be active in a given configuration were obtained. As a final result, only the best SNP per probe is picked, based on the posterior probability for a SNP to be “the” eQTL for a probe. To obtain the posterior probabilities, an estimate of the probability for a probe to have no eQTL in any cell type (π_0_) is needed. This was estimated using Storey and Tibshirani *q*-value approach [58] via probe-level *P*-values obtained by 10,000 permutations.

### Pathway analysis

The Ingenuity Pathway Analysis (IPA) tool was used to establish associations with processes and diseases. The standard IPA enrichment analysis was performed with the set of all human genes on the Illumina array as background.

### Bayesian test for colocalisation

The R package *coloc* [41] was used to assess whether two association signals are consistent with a shared causal variant. Assuming a single causal SNP for both of the traits in a region, posterior support for following hypothesis is estimated:

- H_0_: neither trait has a genetic association in the region;
- H_1_: only *trait*_*1*_ has a genetic association in the region;
- H_2_: only *trait*_*2*_ has a genetic association in the region;
- H_3_: both traits are associated, but with different causal variants;
- H_4_: both traits are associated and share a single causal variant.

For *trait*_*1*_, we chose T1D susceptibility and obtained necessary summary statistics from Onengut-Gumuscu *et al*. [59] from www.t1dbase.org (accessed 13/09/2016) webpage. For *trait*_*2*_, we chose the association with *IRF1* and *STAT1* expression in CD4^+^ T cells. Following Giambartolomei *et al*. [41], we set prior probability (*p*_*1*_) that a variant is associated with *trait*_*1*_ to 10^−4^, prior probability (*p*_*2*_) for *trans*-eQTL effect to 10^−5^, and prior probability (*p*_*12*_) that a variant is associated with both traits to 10^−6^. To show the consistency of the results, we varied *p*_*2*_ and *p*_*12*_ values from 10^−4^ to 10^−5^ and 10^−5^ to 10^−6^, respectively.

### Structural equation modeling (SEM) and mediation analysis

We compared the plausibility of potential models for the effects of the *IL27* SNP on gene expression levels of *STAT1* and *IRF1* using structural equation modeling (SEM). First, to test the model’s concordance to the true population we used the chi-square test. Next, to assess the goodness of fit of the resulting models, we used the following measures: Comparative Fit Index (CFI), Tucker-Lewis Index (TLI) and Root Mean Square Error of Approximation (RMSEA). The CFI and TLI goodness of fit measures indicate good models for values higher than 0.9. RMSEA values less or equal to 0.05 indicate reasonable fit between the model and the data. To compare the models, we used the Akaike information criterion (AIC). Lower values of that theoretic measure indicate better models.

We applied the Sobel test to test whether a mediator carries the influence of the causal variable to the outcome, i.e. testing whether the indirect effect of the causal variable on the outcome via the mediator is significantly different from zero. The test statistic was calculated using the Sobel formula [60]: $ab/\sqrt{b^{2}s_{a}^{2}+a^{2}s_{b}^{2}}$, where *a* is the effect of the causal variable on the mediator and *b* is the effect of the mediator on the outcome and *s*_*a*_ and *s*_*b*_ are the corresponding standard errors of *a* and *b*.

### *IL27-IRF1-STAT1* simulation study

We conducted a simulation study to investigate the relationship between the coding allele of the SNP and expression levels of two genes, *IRF1* and *STAT1*. Therefore, we performed 1000 simulations using a sample size of *n* = 1000. We generated SNP genotypes with the minor allele frequency being the sum of two random binary traits from a binomial distribution *B*(1, 0.37), *IRF1* expression levels depending only on the SNP (*irf*1 = 0.903 − 1.237 × *snp* + *N*(0,1)) and *STAT1* expression levels depending only on *IRF1* expression levels (*stat*1 = 0.031 − 0.534 × *irf*1 + *N*(0,1)). Then using simulated data, we estimated the significance of the SNP allelic effect and *IRF1* expression levels on *STAT1* expression levels and the SNP allelic effect and *STAT1* expression levels on *IRF1* expression levels by linear models.

### Graphics packages

Graphs were generated using R [55] base packages and *ggplot2* [61]. The LD plots were generated using *LDheatmap* [62], the regional association plots were generated with Locus Zoom [63] using hg19/1000 Genomes Nov 2014 EUR for showing LD between the variants, and the circos plots were drawn using the Circos visualization tool [64].

### Statistical analysis

We used linear mixed effects models to estimate the mean of the differences in methylation and expression levels between CD4^+^ and CD8^+^ T cells assessing random intercepts for each of the individuals adjusted for sex, age and batch effects (chip and position on chip). The effect of the mutant SNP on IRF1 and STAT1 transcript levels was also evaluated by linear mixed effects models.

## Supporting information

S1 Fig Estimated replication rate of peripheral blood *cis*-eQTLs in CD4^+^ and CD8^+^ T cells. The histograms show the distribution of *P*-values of significant SNP-probe pairs (probe-level FDR < 0.05) discovered in the meta-analysis by Westra *et al*. [16] in (***A***) CD4^+^ and (***B***) CD8^+^ T cells. The π_1_ statistic [58] estimates the proportion of true positives from the P-value distribution, interpreted as the proportion of replicated *cis*-eQTL effects.
(TIF)

S2 Fig Association between the eQTL effect size and gene expression levels. The scatterplot shows the effect of the “best” SNP allele dosages on gene expression for 4385 significant probes in CD4^+^ T cells (*x*-axis) and in CD8^+^ T cells (*y*-axis). For eQTLs where the absolute effect size was higher in one cell type, there was also a tendency for higher expression levels in the given cell type (chi-square test P-value < 2x10^-16^).
(TIF)

S3 Fig Associations of the *CTLA4* region SNPs with different expression probes in *CTLA4* gene. (***A***) Regional association plots of the *CTLA4* region (chr2:203,736–205,738 Mb) SNPs with association *P*-values for gene expression levels of the three probes in the *CTLA4* gene (1230201, 4010768 and 6400333) in CD4^+^ and CD8^+^ T cells. The SNP rs3087243 is the lead eQTL SNP and is used as the index SNP for showing linkage disequilibrium between the SNPs. (***B***) Heatmap of the correlation coefficients between the T1D and/or RA-associated variants rs231735 [8], rs231806 [28] (MH30C/G), rs3087243 [28,30] (CT60A/G), rs231775 [28] (+49A/G) and gene expression levels of the three different probes in the *CTLA4* gene in CD4^+^ and CD8^+^ T cells. Allele in brackets indicates the assessed allele which is also the MAF allele. (***C***) Linkage disequilibrium (LD) plot for the four T1D and/or RA-associated variants is shown. LD between the SNPs is measured by pairwise r^2^ calculated using the genotypes of 99 individuals from 1000 Genomes project phase 3 CEU population. UCSC genes (based on RefSeq) and their location with respect to SNPs are shown on the top of the LD plot. In the Estonian population, the LD between rs231735 and rs231806, rs3087243 is stronger (r^2^ of 0.85 and 0.76, respectively), the other r^2^ values are similar.
(TIF)

S4 Fig Overview of the chr12q13.2 *trans*-acting locus. (***A***) Expression levels (*y*-axis, quantile normalized and log_2_-transformed) of the six genes in the region by cell type (*x*-axis) are shown by box plots incorporated into violin plots. Violin plot shows the density plot of the data on each side, the lower and upper border of the box correspond to the first and third quartiles, respectively, the central line depicts the median, and whiskers extends from the borders to +/- 1.5xIQR, where IQR stands for inter-quantile range, the distance between the first and third quantiles. (***B***) Linkage disequilibrium (LD) plot for the lead eQTL SNP and eight GWAS SNPs on chromosome 12 *trans*-acting region chr12q13.2 is shown. The GWAS SNPs are linked with their role in disease susceptibility in **Fig 3**. LD between the SNPs is measured by pairwise r^2^ calculated from 99 individuals from 1000 Genomes project phase 3 CEU population. UCSC genes (based on RefSeq) and their location with respect to SNPs are shown on the top of the LD plot. There is strong LD (r^2^ > 0.8) between the lead eQTL SNP rs1131017 and three GWAS SNPs rs10876864, rs11171739 and rs773125 (r^2^ of 1.00, 0.92 and 0.88, respectively, in CEU population and r^2^ of 0.98, 0.98 and 0.84, respectively, in the Estonian population). (***C***) Regional association plots including all SNPs at chr12q13.2. The smallest association *P*-values for the SNPs are shown in CD4^+^ and CD8^+^ T cells. The SNP rs1131017 is the lead SNP in the region with the smallest *P*-value, and the eight GWAS SNPs are highlighted.
(TIF)

S5 Fig Structure model of the human IL-27 (Q8NEV9; residues 61 to 134, based on http://www.proteinmodelportal.org/) and location of the amino acid lysine (L119) that is changed to proline (P119) by the rs181206 missense mutation. We identified a common missense variant rs181206[A/G] in cytokine *IL27* as a *trans*-eQTL for *IRF1* and *STAT1* in CD4^+^ T cells. The G allele of the variant alters an amino acid in the alpha-helical domain from leucine to proline at position 119.
(TIF)

S6 Fig Associations of the *IL27* region SNPs with *IRF1* and *STAT1* gene expression levels in CD8^+^ T cells, B cells and in monocytes. Regional association plots of the *IL27* region (chr16:28,2–29,1 Mb) SNPs with association *P*-values for (***A***) *IRF1* (chr5) and (***B***) *STAT1* (chr2) gene expression levels in CD8^+^ T cells, B cells, and monocytes. The SNP rs181206 is used as the index SNP for showing linkage disequilibrium between the SNPs.
(TIF)

S7 Fig Associations of the *IL27* region SNPs with *IL27* gene expression levels in CD4^+^ T cells, CD8^+^ T cells, B cells and in monocytes. Regional association plots of the *IL27* region (chr16:28,2–29,1 Mb) SNPs with association *P*-values for the *IL27* gene expression levels in CD4^+^ T cells, CD8^+^ T cells, B cells, and monocytes. The SNP rs181206 is used as the index SNP for showing linkage disequilibrium between the SNPs.
(TIF)

S8 Fig Comparison of two models IRF ~ STAT1 + SNP and STAT1 ~ IRF1 + SNP on observed and simulated data. We performed 1000 simulations using a sample size of *n* = 1000. We generated SNP genotypes, *IRF1* and *STAT1* expression levels according to the plausible causal model 1) SNP -> *IRF1* -> *STAT1* as follows: we generated SNP genotypes with minor allele frequency as the sum of a two random binary traits from binomial distribution *B*(1, 0.37), *IRF1* expression levels depending only on the SNP (*irf1 = 0*.*903–1*.*237* x *snp + N(0*,*1)*) and *STAT1* expression levels depending only on *IRF1* expression levels (*stat1 = 0*.*031 + 0*.*534* x *irf1 + N(0*,*1)*). Then using simulated and observed data, we compared the estimates obtained from two linear models IRF ~ STAT1 + SNP (upper panel) and STAT1 ~ IRF1 + SNP (lower panel). Parameter estimates with 95% confidence intervals are shown for every explanatory variable for observed and simulation data. A similar pattern of the parameter estimates supports the validity of causal model 1).
(TIF)

S9 Fig A scheme on IL-27 role in activation of *IRF1* and *STAT1* gene expression. IL27 (as a heterodimer with EBI3), upon binding to its receptor, activates the STAT1/STAT3 pathway. After binding to interferon stimulated response elements (ISRE), STAT1/STAT3 pathway induces transcription of several interferon-induced genes, including *IRF1* and *STAT1* itself. IRF1 is a transcription factor that enhances the expression of *STAT1* gene. We identified a common missense variant in cytokine *IL27* as a *trans*-eQTL for *IRF1* and *STAT1* in CD4^+^ T cells. Our model suggests that *IRF1* mediates the SNP and *STAT1* relationship. Moreover, our functional studies with the mutated form of IL-27 (that is associated with protection against T1D via linkage disequilibrium with GWAS SNP rs4788084) confirmed its decreased capacity to activate the STAT1 pathway.
(TIF)

S1 Table Significant *cis*-eQTL effects in CD4^+^ and CD8^+^ T cells (probe-level false discovery rate < 0.05). (XLSX)

S2 Table Significant results of multi-tissue joint analysis (FDR < 0.05). Bayesian model averaging and hierarchical modelling framework by Flutre *et al*. was used implemented in the eQtlBma program. Only the best SNP per probe is picked, based on the posterior probability for a SNP to be “the” eQTL for that probe. Posteriors of all configurations together with summary statistics in CD4^+^ and CD8^+^ T cells are reported. There are 4385 probes with eQTLs within 3871 genes (FDR < 0.05).
(XLSX)

S3 Table Significant differences in DNA methylation levels between CD4^+^ and CD8^+^ T cells among genes with seemingly isoform-specific effects between the two cell types. There were 408 out of 3,024 genes affected by a SNP which were covered by more than one expression probe with *cis*-eQTL effects in CD4^+^ and/or CD8^+^ T cells. We could find CpG sites in the proximity (+/- 1000 basepairs) of the gene for 405 genes covered by 8665 CpG sites. We tested those CpG sites for differential methylation between CD4^+^ and CD8^+^ T cells. The effect on the mean of the differences in CD8^+^ compared to CD4^+^ T cells was evaluated using linear mixed models. False discovery rate based multiple testing was used based on all tested CpG sites. CpG sites with the most significant differences in methylation are listed per 74 gene out of 187 with seemingly isoform-specific effects in the tissue-by-tissue analysis. Multi-tissue analysis could find eQTL effect for two or more probes in 66 genes (of the 74 genes) with high posterior confidence to be shared between the two cell types.
(XLS)

S4 Table Significant *trans*-eQTL effects with SNPs associated with human diseases or traits in CD4^+^ and CD8^+^ T cells (probe-level false discovery rate < 0.05). (XLS)

S5 Table Significant *trans*-eQTL effects with SNPs at chr12q13.2 in CD4^+^ and CD8^+^ T cells (probe-level false discovery rate < 0.05). (XLS)

S6 Table *IL27* region *trans*-eQTL mapping results for the *IRF1* and *STAT1* genes in CD4^+^ T cells, CD8^+^ T cells, monocytes, and B cells. (XLS)

S7 Table T1D/eQTL colocalisation. Results of the colocalisation analysis between the *trans*-eQTLs for *IRF1* (A) and *STAT1* (B) in CD4^+^ T cells and the type 1 diabetes (T1D) susceptibility using different prior probabilities. The columns “*T1D pval*” and “*eQTL pval*” note the lowest *P*-value found for the association with T1D from Onengut-Gumuscu *et al*. study using T1DBase database (www.t1dbase.org), and for the expression association in CD4^+^ T cells respectively, with the corresponding SNP name (“*T1D SNP*” and “*eQTL SNP*”), “*Best Causal*” reports the SNP with the highest posterior probability to be the true causal variant among the two. Different prior probabilities for observing *trans*-eQTL effect (*p*_*2*_) and different prior probabilities for the variant being associated with both traits (*p*_*12*_) are used to estimate the poster probability for different signal (different causal variant for associated traits, *PP3*) and common signal (shared single causal variant for associated traits, *PP4*).
(DOCX)

S8 Table SEM fit statistics for three alternative causal models. To test the model’s concordance to true population we used chi-square test. To assess the goodness of fit of the resulting models, we used the following measures: Comparative Fit Index (CFI), Tucker-Lewis Index (TLI) and Root Mean Square Error of Approximation (RMSEA). The CFI and TLI goodness of fit measures indicate good models for values higher than 0.9. RMSEA values less or equal to 0.05 indicate reasonable fit between the model and the data. To compare models we used the Akaike information criterion (AIC). Lower values of the theoretic measures indicate better models.
(DOCX)

S1 File Supplementary material and methods. (PDF)
