# Supplementary figures and images for: Pathogenic implications for autoimmune mechanisms derived by comparative eQTL analysis of CD4+ versus CD8+ T cells
Source: PLoS Genet. 2017 Mar 1;13(3):e1006643. doi: 10.1371/journal.pgen.1006643 (PMC5352142; doi:10.1371/journal.pgen.1006643)

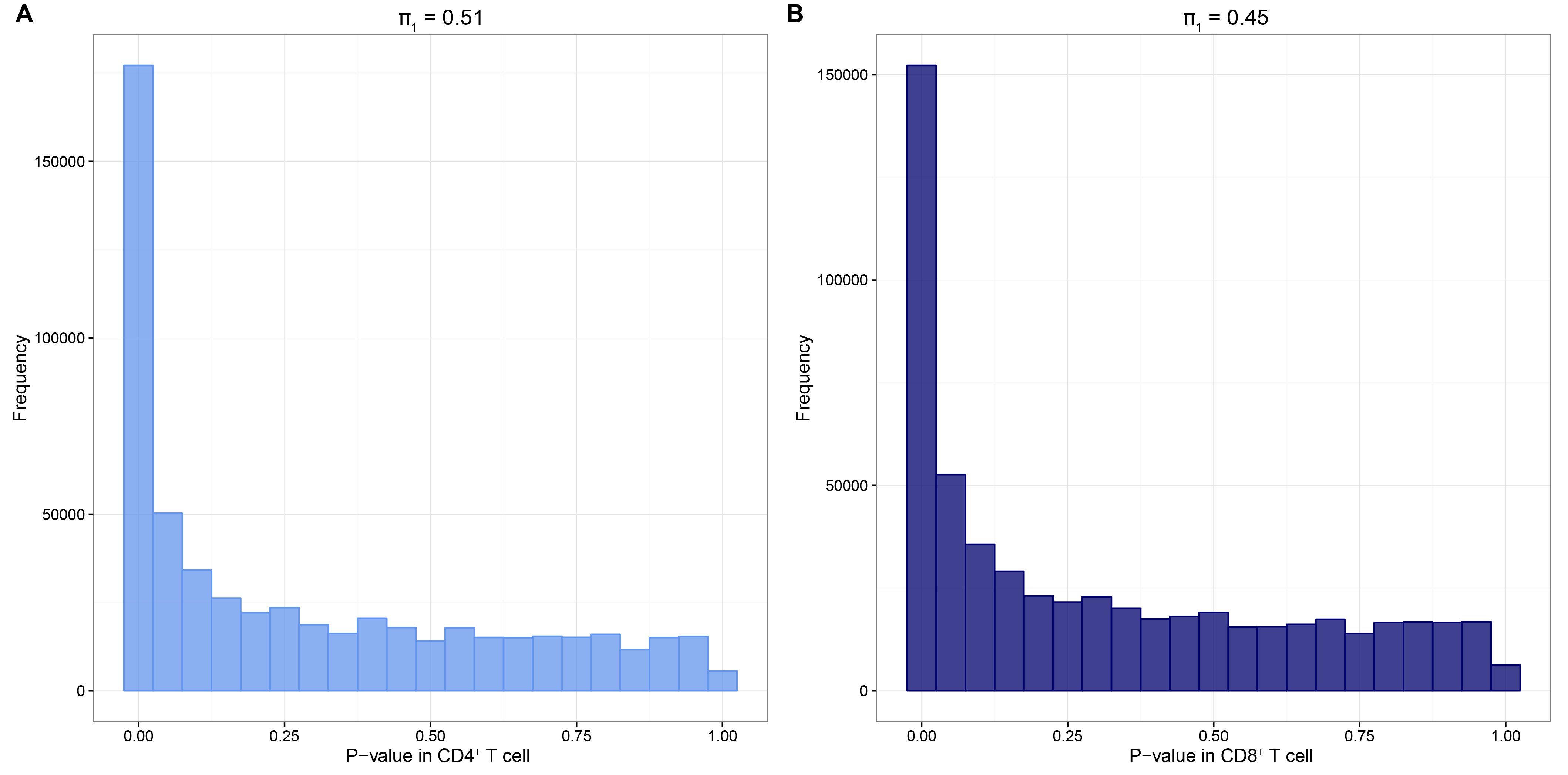

Supplement: S1 Fig — The histograms show the distribution of P-values of significant SNP-probe pairs (probe-level FDR < 0.05) discovered in the meta-analysis by Westra et al. [16] in (A) CD4+ and (B) CD8+ T cells. The π1 statistic [58] estimates the proportion of true positives from the P-value distribution, interpreted as the proportion of replicated cis-eQTL effects. (TIF) [file pgen.1006643.s001.tif]

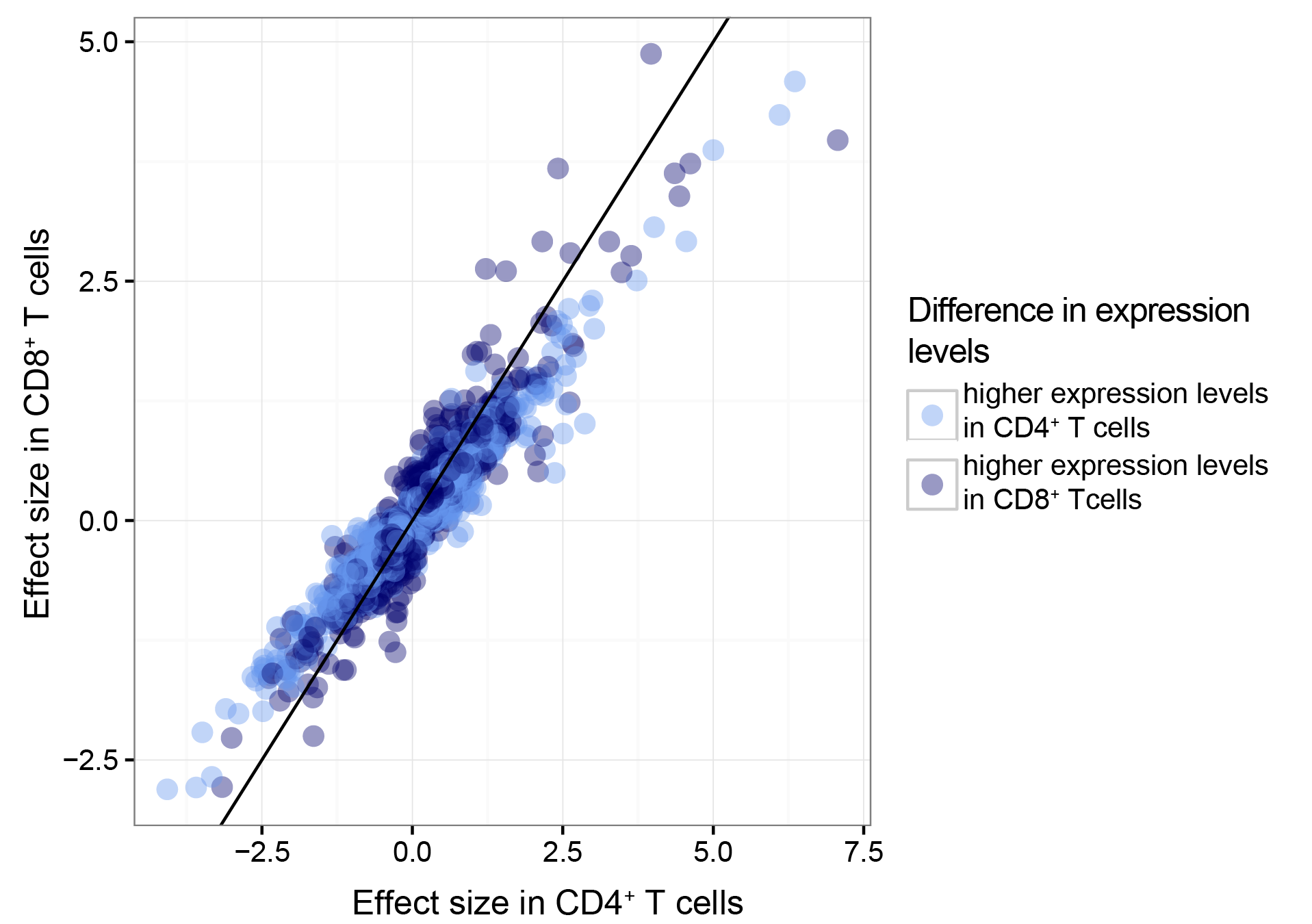

Supplement: S2 Fig — The scatterplot shows the effect of the “best” SNP allele dosages on gene expression for 4385 significant probes in CD4+ T cells (x-axis) and in CD8+ T cells (y-axis). For eQTLs where the absolute effect size was higher in one cell type, there was also a tendency for higher expression levels in the given cell type (chi-square test P-value < 2x10^-16^). (TIF) [file pgen.1006643.s002.tif]

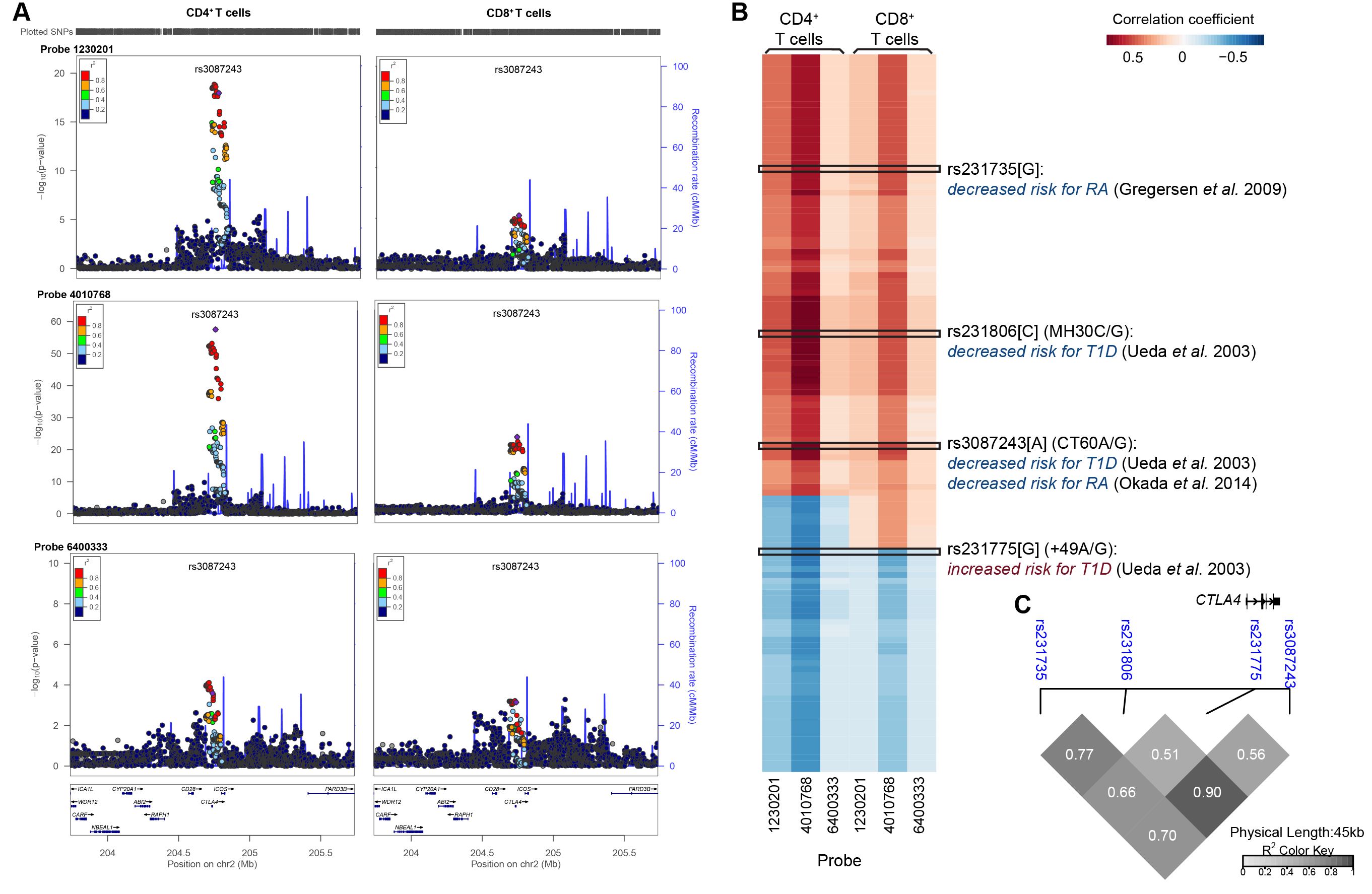

Supplement: S3 Fig — (A) Regional association plots of the CTLA4 region (chr2:203,736–205,738 Mb) SNPs with association P-values for gene expression levels of the three probes in the CTLA4 gene (1230201, 4010768 and 6400333) in CD4+ and CD8+ T cells. The SNP rs3087243 is the lead eQTL SNP and is used as the index SNP for showing linkage disequilibrium between the SNPs. (B) Heatmap of the correlation coefficients between the T1D and/or RA-associated variants rs231735 [8], rs231806 [28] (MH30C/G), rs3087243 [28,30] (CT60A/G), rs231775 [28] (+49A/G) and gene expression levels of the three different probes in the CTLA4 gene in CD4+ and CD8+ T cells. Allele in brackets indicates the assessed allele which is also the MAF allele. (C) Linkage disequilibrium (LD) plot for the four T1D and/or RA-associated variants is shown. LD between the SNPs is measured by pairwise r2 calculated using the genotypes of 99 individuals from 1000 Genomes project phase 3 CEU population. UCSC genes (based on RefSeq) and their location with respect to SNPs are shown on the top of the LD plot. In the Estonian population, the LD between rs231735 and rs231806, rs3087243 is stronger (r2 of 0.85 and 0.76, respectively), the other r2 values are similar. (TIF) [file pgen.1006643.s003.tif]

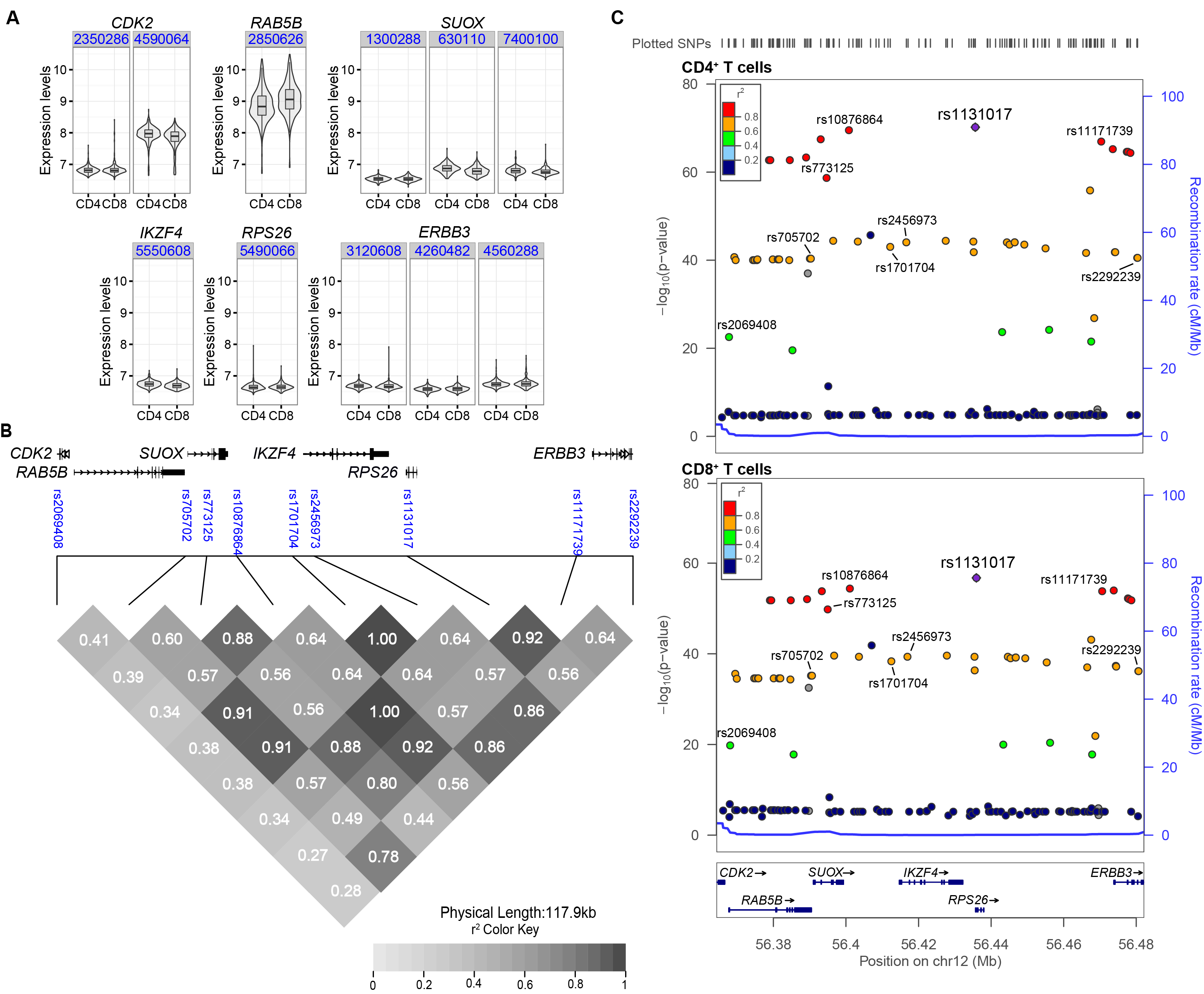

Supplement: S4 Fig — (A) Expression levels (y-axis, quantile normalized and log2-transformed) of the six genes in the region by cell type (x-axis) are shown by box plots incorporated into violin plots. Violin plot shows the density plot of the data on each side, the lower and upper border of the box correspond to the first and third quartiles, respectively, the central line depicts the median, and whiskers extends from the borders to +/- 1.5xIQR, where IQR stands for inter-quantile range, the distance between the first and third quantiles. (B) Linkage disequilibrium (LD) plot for the lead eQTL SNP and eight GWAS SNPs on chromosome 12 trans-acting region chr12q13.2 is shown. The GWAS SNPs are linked with their role in disease susceptibility in Fig 3. LD between the SNPs is measured by pairwise r2 calculated from 99 individuals from 1000 Genomes project phase 3 CEU population. UCSC genes (based on RefSeq) and their location with respect to SNPs are shown on the top of the LD plot. There is strong LD (r2 > 0.8) between the lead eQTL SNP rs1131017 and three GWAS SNPs rs10876864, rs11171739 and rs773125 (r2 of 1.00, 0.92 and 0.88, respectively, in CEU population and r2 of 0.98, 0.98 and 0.84, respectively, in the Estonian population). (C) Regional association plots including all SNPs at chr12q13.2. The smallest association P-values for the SNPs are shown in CD4+ and CD8+ T cells. The SNP rs1131017 is the lead SNP in the region with the smallest P-value, and the eight GWAS SNPs are highlighted. (TIF) [file pgen.1006643.s004.tif]

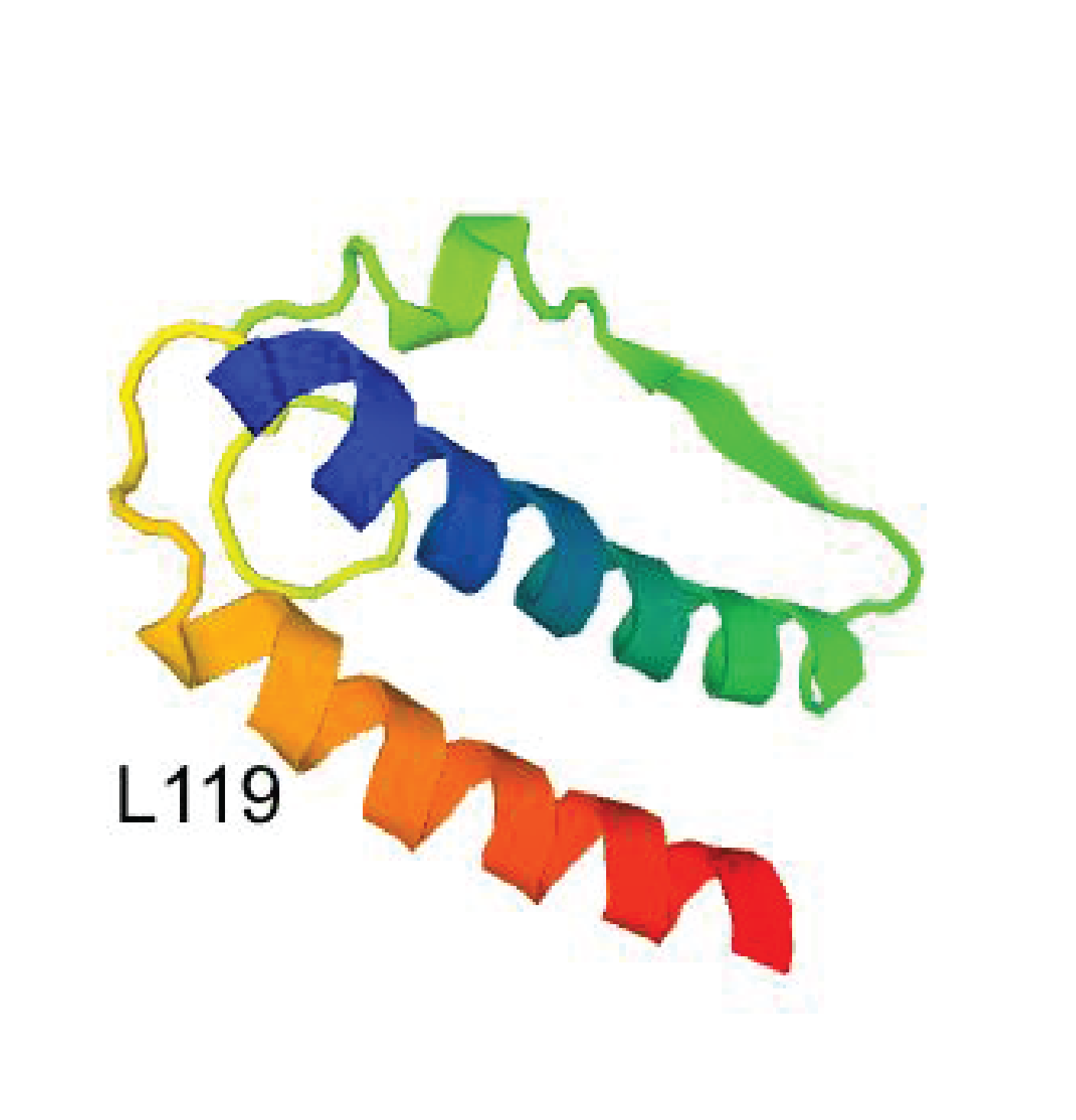

Supplement: S5 Fig — We identified a common missense variant rs181206[A/G] in cytokine IL27 as a trans-eQTL for IRF1 and STAT1 in CD4+ T cells. The G allele of the variant alters an amino acid in the alpha-helical domain from leucine to proline at position 119. (TIF) [file pgen.1006643.s005.tif]

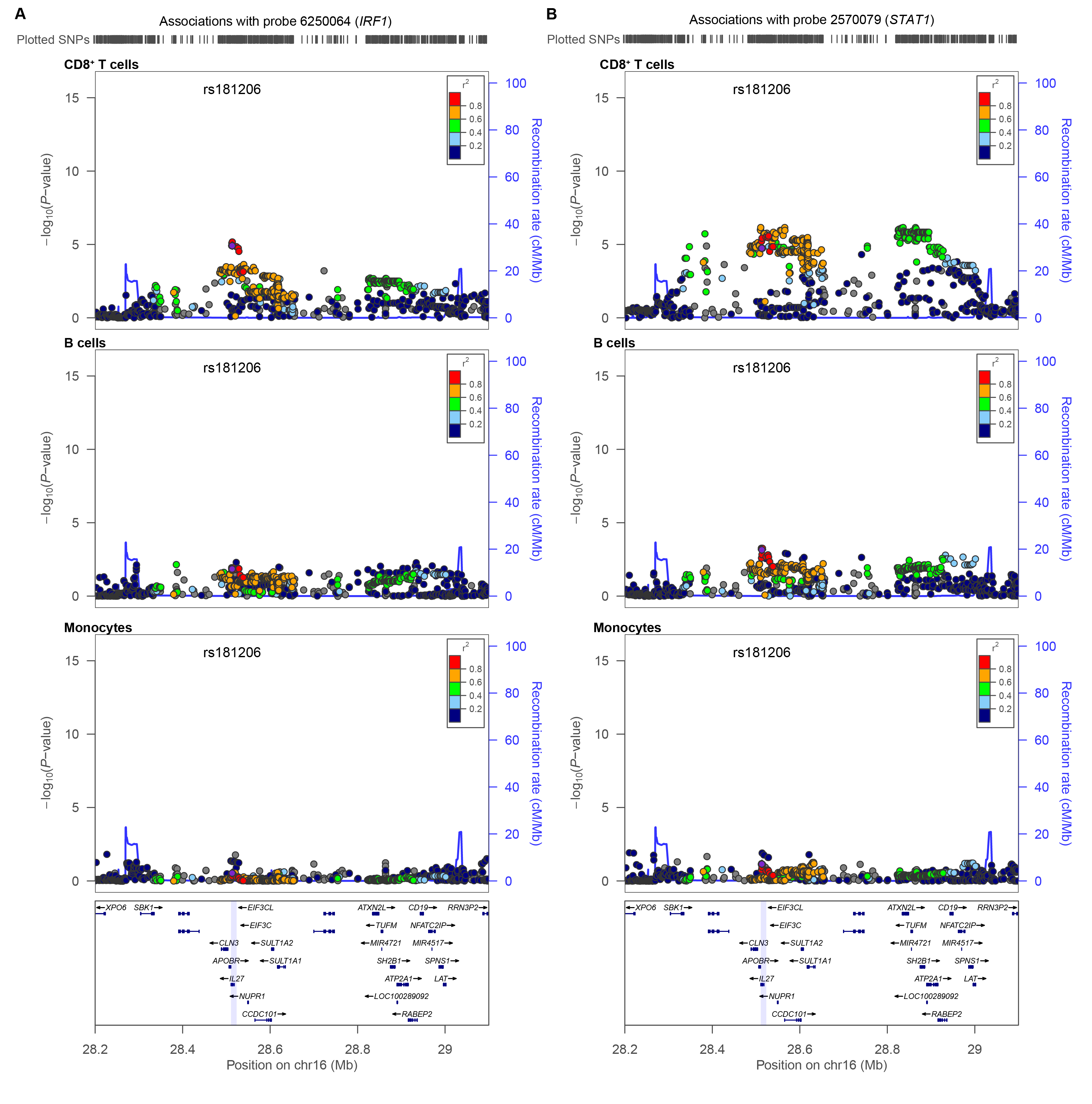

Supplement: S6 Fig — Regional association plots of the IL27 region (chr16:28,2–29,1 Mb) SNPs with association P-values for (A) IRF1 (chr5) and (B) STAT1 (chr2) gene expression levels in CD8+ T cells, B cells, and monocytes. The SNP rs181206 is used as the index SNP for showing linkage disequilibrium between the SNPs. (TIF) [file pgen.1006643.s006.tif]

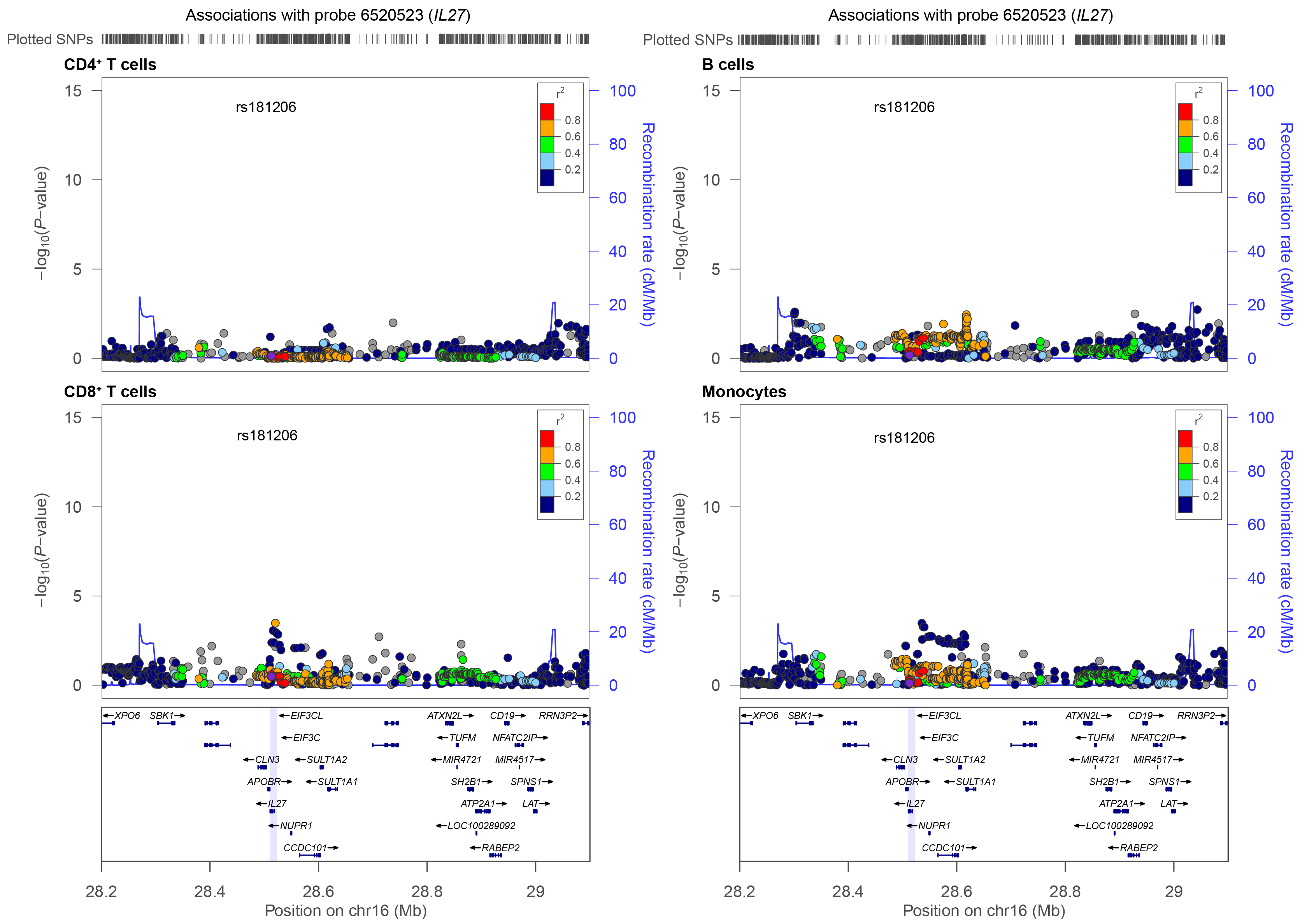

Supplement: S7 Fig — Regional association plots of the IL27 region (chr16:28,2–29,1 Mb) SNPs with association P-values for the IL27 gene expression levels in CD4+ T cells, CD8+ T cells, B cells, and monocytes. The SNP rs181206 is used as the index SNP for showing linkage disequilibrium between the SNPs. (TIF) [file pgen.1006643.s007.tif]

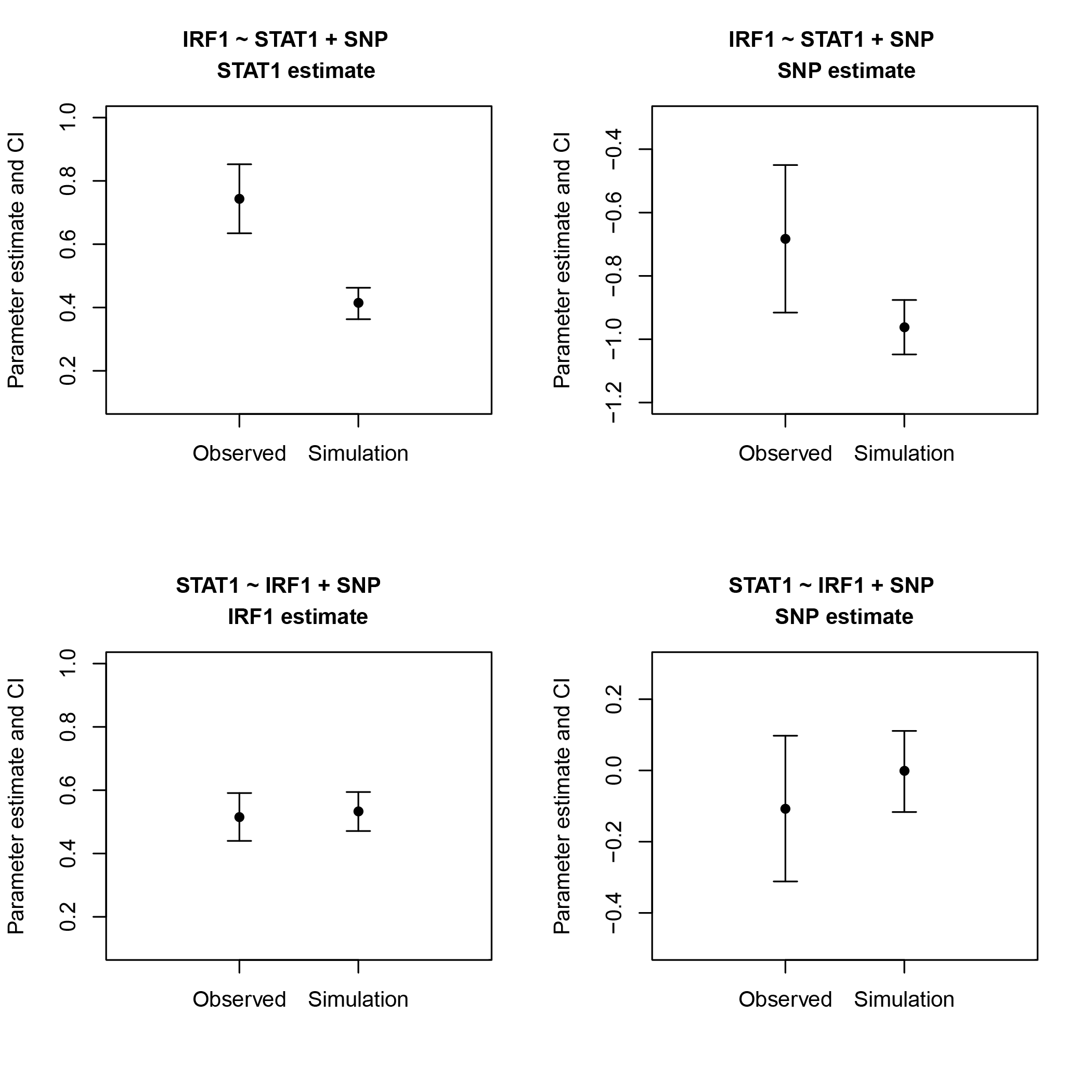

Supplement: S8 Fig — We performed 1000 simulations using a sample size of n = 1000. We generated SNP genotypes, IRF1 and STAT1 expression levels according to the plausible causal model 1) SNP -> IRF1 -> STAT1 as follows: we generated SNP genotypes with minor allele frequency as the sum of a two random binary traits from binomial distribution B(1, 0.37), IRF1 expression levels depending only on the SNP (irf1 = 0.903–1.237 x snp + N(0,1)) and STAT1 expression levels depending only on IRF1 expression levels (stat1 = 0.031 + 0.534 x irf1 + N(0,1)). Then using simulated and observed data, we compared the estimates obtained from two linear models IRF ~ STAT1 + SNP (upper panel) and STAT1 ~ IRF1 + SNP (lower panel). Parameter estimates with 95% confidence intervals are shown for every explanatory variable for observed and simulation data. A similar pattern of the parameter estimates supports the validity of causal model 1). (TIF) [file pgen.1006643.s008.tif]

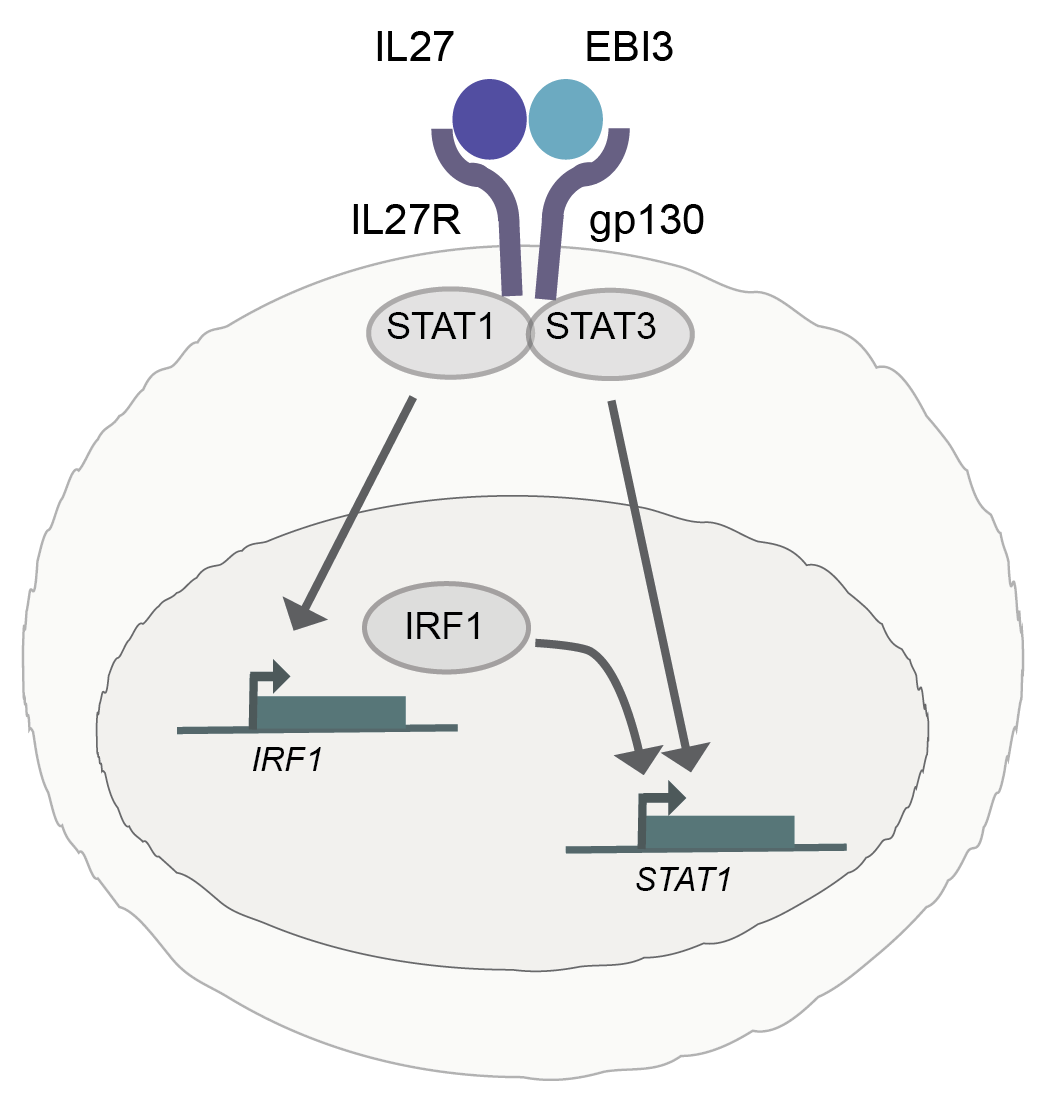

Supplement: S9 Fig — IL27 (as a heterodimer with EBI3), upon binding to its receptor, activates the STAT1/STAT3 pathway. After binding to interferon stimulated response elements (ISRE), STAT1/STAT3 pathway induces transcription of several interferon-induced genes, including IRF1 and STAT1 itself. IRF1 is a transcription factor that enhances the expression of STAT1 gene. We identified a common missense variant in cytokine IL27 as a trans-eQTL for IRF1 and STAT1 in CD4+ T cells. Our model suggests that IRF1 mediates the SNP and STAT1 relationship. Moreover, our functional studies with the mutated form of IL-27 (that is associated with protection against T1D via linkage disequilibrium with GWAS SNP rs4788084) confirmed its decreased capacity to activate the STAT1 pathway. (TIF) [file pgen.1006643.s009.tif]
