# Supplementary material for: Pathogenic implications for autoimmune mechanisms derived by comparative eQTL analysis of CD4+ versus CD8+ T cells
Source: PLoS Genet. 2017 Mar 1;13(3):e1006643. doi: 10.1371/journal.pgen.1006643 (PMC5352142; doi:10.1371/journal.pgen.1006643)
Supplement: S7 Table — Results of the colocalisation analysis between the trans-eQTLs for IRF1 (A) and STAT1 (B) in CD4+ T cells and the type 1 diabetes (T1D) susceptibility using different prior probabilities. The columns “T1D pval” and “eQTL pval” note the lowest P-value found for the association with T1D from Onengut-Gumuscu et al. study using T1DBase database (www.t1dbase.org), and for the expression association in CD4+ T cells respectively, with the corresponding SNP name (“T1D SNP” and “eQTL SNP”), “Best Causal” reports the SNP with the highest posterior probability to be the true causal variant among the two. Different prior probabilities for observing trans-eQTL effect (p2) and different prior probabilities for the variant being associated with both traits (p12) are used to estimate the poster probability for different signal (different causal variant for associated traits, PP3) and common signal (shared single causal variant for associated traits, PP4). (DOCX) [file pgen.1006643.s016.docx]

**S7 Table. T1D/eQTL colocalisation.** Results of the colocalisation analysis between the *trans*-eQTLs for *IRF1* (A) and *STAT1* (B) in CD4^+^ T cells and the type 1 diabetes (T1D) susceptibility using different prior probabilities. The columns “*T1D pval*” and “*eQTL pval*” note the lowest p-value found for the association with T1D from Onengut-Gumuscu *et al.* study using T1DBase database (www.t1dbase.org), and for the expression association in CD4^+^ T cells respectively, with the corresponding SNP name (“*T1D SNP*” and “*eQTL SNP*”), “*Best Causal*” reports the SNP with the highest posterior probability to be the true causal variant among the two. Different prior probabilities for observing *trans-*eQTL effect (*p_2_*) and different prior probabilities for the variant being associated with both traits (*p_12_*) are used to estimate the poster probability for different signal (different causal variant for associated traits, *PP3*) and common signal (shared single causal variant for associated traits, *PP4*).

| **A) *IRF1*** |  |  |  |  |  |  |  |  |  |  |  |  |  |  |
| --- | --- | --- | --- | --- | --- | --- | --- | --- | --- | --- | --- | --- | --- | --- |
| **Region** | **Gene** | **T1D**  **pval** | **T1D**  **SNP** | **eQTL pval** | **eQTL SNP** | **Best Causal** | **p_2_ = 10^-4^ &  p_12_ = 10^-5^** | | **p_2_ = 10^-4^ &  p_12_ = 10^-6^** | | **p_2_ = 10^-5^ &  p_12_ = 10^-5^** | | **p_2_ = 10^-5^ &  p_12_ = 10^-6^** | |
|  |  |  |  |  |  |  | *PP3* | *PP4* | *PP3* | *PP4* | *PP3* | *PP4* | *PP3* | *PP4* |
| 16p11.2 | IL27 | 1.03E-09 | rs4788084 | 3.69E-16 | rs181206 | rs181206 | 1.0% | 99.0% | 9.3% | 90.7% | 0.1% | 99.9% | 1.0% | 99.0% |
|  |  |  |  |  |  |  |  |  |  |  |  |  |  |  |
| **B) *STAT1*** | |  |  |  |  |  |  |  |  |  |  |  |  |  |
| **Region** | **Gene** | **T1D**  **pval** | **T1D**  **SNP** | **eQTL pval** | **eQTL SNP** | **Best Causal** | **p_2_ = 10^-4^ &  p_12_ = 10^-5^** | | **p_2_ = 10^-4^ &  p_12_ = 10^-6^** | | **p_2_ = 10^-5^ &  p_12_ = 10^-5^** | | **p_2_ = 10^-5^ &  p_12_ = 10^-6^** | |
|  |  |  |  |  |  |  | *PP3* | *PP4* | *PP3* | *PP4* | *PP3* | *PP4* | *PP3* | *PP4* |
| 16p11.2 | IL27 | 1.03E-09 | rs4788084 | 1.90E-10 | rs181206 | rs181206 | 0.7% | 99.3% | 6.8% | 93.1% | 0.1% | 99.9% | 0.7% | 99.2% |
|  |  |  |  |  |  |  |  |  |  |  |  |  |  |  |
