# Supplementary material for: Pathogenic implications for autoimmune mechanisms derived by comparative eQTL analysis of CD4+ versus CD8+ T cells
Source: PLoS Genet. 2017 Mar 1;13(3):e1006643. doi: 10.1371/journal.pgen.1006643 (PMC5352142; doi:10.1371/journal.pgen.1006643)
Supplement: S8 Table — To test the model’s concordance to true population we used chi-square test. To assess the goodness of fit of the resulting models, we used the following measures: Comparative Fit Index (CFI), Tucker-Lewis Index (TLI) and Root Mean Square Error of Approximation (RMSEA). The CFI and TLI goodness of fit measures indicate good models for values higher than 0.9. RMSEA values less or equal to 0.05 indicate reasonable fit between the model and the data. To compare models we used the Akaike information criterion (AIC). Lower values of the theoretic measures indicate better models. (DOCX) [file pgen.1006643.s017.docx]

**S8 Table. SEM fit statistics for three alternative causal models.**

| **Fit statistics** | **Model (1) SNP -> *IRF1* –> *STAT1*** | **Model (2) SNP -> *STAT1* -> *IRF1*** | **Model (3) *STAT1* <- SNP -> *IRF1*** |
| --- | --- | --- | --- |
| Chisq (df); *P*-value of Model-implied-covariance = Sample covariance | 1.072 (1); 0.301 | 31.839 (1);  0.000 | 141.605 (1);  0.000 |
| AIC | 2664.044 | 2694.811 | 2804.576 |
| CFI | 1.000 | 0.874 | 0.426 |
| TLI | 0.999 | 0.623 | -0.721 |
| RMSEA (CI);  *P*-value of RMSEA ≤ 0.05 | 0.016 (0, 0.157); 0.458 | 0.324 (0.234, 0.426); 0.000 | 0.693 (0.599, 0.791); 0.000 |
