# Supplementary material for: Pathogenic implications for autoimmune mechanisms derived by comparative eQTL analysis of CD4+ versus CD8+ T cells
Source: PLoS Genet. 2017 Mar 1;13(3):e1006643. doi: 10.1371/journal.pgen.1006643 (PMC5352142; doi:10.1371/journal.pgen.1006643)
Supplement: S1 File — (PDF) [file pgen.1006643.s018.pdf]

Supplementary Materials and Methods for

Pathogenic Implications for Autoimmune Mechanisms Derived by Comparative eQTL Analysis of CD4$^+$ Versus CD8$^+$ T cells

Silva Kasela, Kai Kisand, Liina Tserel, Epp Kaleviste, Anu Remm, Krista Fischer, Tõnu Esko, Harm-Jan Westra, Benjamin P Fairfax, Seiko Makino, Julian C Knight, Lude Franke, Andres Metspalu, Pärt Peterson*, Lili Milani*

*Corresponding author. E-mail: part.peterson@ut.ee (P.P.); lili.milani@ut.ee (L.M.)

Supplementary material and methods

Genotyping and imputation

DNA from the samples were genotyped using HumanOmniExpress BeadChips (Illumina), according to the manufacturer's instructions. The dataset was imputed using the 1000 Genomes project reference by using IMPUTE v2(1).

Directly genotyped SNPs were coded as 0, 1 or 2 and for imputed SNPs we used allele dosage values that ranged between 0 and 2. SNPs with imputation quality lower than 0.4 were removed from the analysis. A final set of 5,879,386 autosomal SNPs with a minor allele frequency (MAF) of > 0.05 was used for further analysis.

Purification of $CD4^+$ and $CD8^+$ T cells

Peripheral blood was obtained from healthy donors of the Estonian Genome Center of the University of Tartu. Peripheral blood mononuclear cells (PBMC) were extracted using Ficoll-Paque (GE Healthcare) gradient centrifugation. $CD4^+$ and $CD8^+$ T cells were extracted from the PBMCs by consecutive positive separation using microbeads ($CD4^+$ #130-045-101; $CD8^+$ #130-045-201) and AutoMACS technology (Miltenyi Biotec) according to the manufacturer's protocol. The purity of the extracted $CD4^+$ T cells was 91–95% and for $CD8^+$ T cells 88–91%. All cell populations were collected and stored as cell pellets in a −80 °C freezer.

DNA extraction, bisulfite treatment, DNA methylation measurement and normalization

DNA extraction, bisulfite treatment, measurements, and normalization of DNA methylation data has been described before in Tserel *et al.*(2) and the data is stored at NCBI Gene Expression Omnibus (http://www.ncbi.nlm.nih.gov/geo/) with the accession number GSE59065. In total, DNA methylation levels in $CD4^+$ were available for 97 samples and DNA methylation levels in $CD8^+$ T cells were available for 99 samples.

RNA extraction, labelling and hybridization

RNA was extracted from the purified T cells using the miRNeasy Mini Kit combined with a recommended RNase-free DNase I treatment (both from Qiagen) according to the manufacturer's protocol. RNA from whole blood was purified using the MagMAX™ for Stabilized Blood Tubes RNA Isolation Kit. RNA was concentrated using the Heraeus vacuum centrifugation system without heating. The RNA was labeled and amplified using the

TargetAmp Nano Labeling Kit for Illumina Expression BeadChip (Epicentre Biotechnologies) with SuperScript III Reverse *Trans*criptase (Life Technologies) according to the manufacturer's protocol, followed by purification with the RNeasy MinElute Cleanup Kit (Qiagen). RNA quality was assessed after extraction and after labelling using an Agilent 2100 Bioanalyzer and Agilent RNA 6000 Nano Kit (all from Agilent Technologies). The labelled RNA samples were hybridized to HumanHT12-v4 Expression BeadChips (Illumina) according to the manufacturer's instructions.

Gene expression probe mapping

Illumina HT12-v4 BeadChip probe sequences were mapped to the human genome build GRCh37 using TopHat(3) allowing up to five mismatches. Transcript and genome information was retrieved from Ensembl for genome assembly GRCh37.p13. Expression probes that mapped uniquely to the reference with no mismatches were retained. All expression probes mapping to either the X or Y chromosomes were removed. A total of 38,839 expression probes remained for further analyses.

Gene expression quality control and normalization

Preprocessing and quality control of the data was done using R(4) and the Bioconductor packages *lumi*(5) and *arrayQualityMetrics*(6). We chose to be conservative on determining outliers. Therefore, we excluded samples 1) which were considered to be outliers based on at least two criteria (array intensity distribution and/or between array comparison and/or individual array quality) in microarray quality metrics reports; 2) with detection rate (using detection *P*-value cut-off 0.05) less than 10% in $CD4^+$ and $CD8^+$ T cell samples; 3) which were outliers based on *XIST* gene and Y chromosome expression patterns. Also, mix-ups between gene expression samples and genotype samples were corrected using *MixupMapper*(7). The number of samples retained for further analysis was 293 for $CD4^+$ and 283 for $CD8^+$ T cells, 303 unique individuals. Based on similar studies(8), sample size of 300 individuals is sufficient to ensure statistical power to detect eQTL effects in purified cells.

Further, gene expression data was quantile normalized to the median distribution, and then $\log_2$ transformed. Then separately, for both cell types, probe and sample means were centered to zero and scaled. Correction for possible population structure in the gene expression data was done by removing four multi-dimensional scaling components (MDS components obtained from genotype data using PLINK(9)) using linear regression. Next, principal component analysis (PCA) was conducted on the sample correlation matrix to

correct for environmental and experimental variation. The optimal number of PCs to remove was determined based on the maximum number of *cis*-eQTLs (described before in Westra *et al.*(10)). Only PCs that were not affected by genetic variants (did not show significance at the FDR threshold of 0) were used to correct the gene expression data to minimize the amount of genetic variation removed. The optimal number of PCs to remove was 30 in $CD4^+$ and 45 in $CD8^+$ T cell datasets.

Biological effect of mutant and wild-type *IL27*

Coding sequences of IL27A (wild-type and mutant) and EBI3 without signal peptides were cloned into a modified pPK-CMV-F4 fusion vector (PromoCell GmbH, Heidelberg; Germany) downstream of the naturally secreted Gaussia luciferase (Gluc) that was cloned into the plasmid instead of the Firefly luciferase. HEK 293 cells were transfected with cloned constructs and the secreted Gluc-antigen fusion protein was collected with the tissue culture supernatant 48 hours later. The amount of secreted cytokine in the harvested culture supernatant was quantified according to the luminescence signal that was recorded after the addition of Gluc substrate (coelenterazine GAR-2B, Targeting Systems, CA, USA). Then, 150 million luciferase units (LU) of IL27A (wild-type or mutant) was combined with the same amount of EBI3 to stimulate PBMCs (0.5 million cells per well in a 24-well plate) isolated from four healthy persons, of them one person was used in two parallel sets, for 5 hours at 37°C 5% CO_2. The culture supernatant collected from the HEK cells transfected with a control Gluc vector was used for non-stimulated control PBMCs. RNA was isolated from the stimulated PBMCs using the RNeasy Micro kit (Qiagen), and reverse-transcribed using Superscript III (Invitrogen), 10mM dNTP Mix, RiboLock RNase inhibitor and random hexamers (Thermo Scientific). Real time quantitative PCR (qPCR) was performed using the Applied Biosystems® ViiA™ 7 Real-Time PCR System with a 384-Well Block (Life Technologies) and the Maxima SYBR Green /ROX qPCR Master Mix (Thermo Scientific). Every sample was run in 3 parallel reactions. Relative transcript levels were calculated using the comparative C_t ($\Delta\Delta C_t$) method, and expressed as $2^{-\Delta\Delta C_t}$, where C_t represents the threshold cycle. Primers for *STAT1* and *IRF1* were designed to span the area that is covered by the Illumina gene expression probe and *ACTB* was used as a housekeeping gene. Primers used: *STAT1* forward ATGCACACAGTGCTTTCCGT, reverse ACAGCTAAACCTCAGCAATTAGA; *IRF1* forward GAGCTCTTGGCCTTTGCATTT, reverse CCAATTTTAAGTCATACCAAGGCG; *ACTB* forward CTGGAACGGTGAAGGTGACA; reverse CGGCCACATTGTGAACTTTG.

Cis- and *trans*-eQTL mapping

The effects of SNPs on local (*cis*-eQTL) and distant (*trans*-eQTL) genes were determined via eQTL mapping as described in eQTL mapping analysis cookbook developed by the University Medical Center Groningen at the Genetics Department and the Genomics Coordination Center (https://github.com/molgenis/systemsgenetics/wiki/eQTL-mapping-analysis-cookbook) and in Westra *et al.*(10). For *cis*-analysis the distance between the probe midpoint and SNP genomic location was up to 1 Mb and for *trans*-analysis the distance was more than 5 Mb or the probe and SNP were on different chromosomes. Only SNPs with a MAF > 0.05, call rate > 0.95 and a Hardy-Weinberg equilibrium *P*-value > 0.001 were included in the analyses.

For *cis*-analysis, we only tested SNP-probe pairs if the SNP was not in linkage disequilibrium (LD, $r^2 > 0.2$) with any other SNP in the probe sequence, using the same dataset to determine the effects, in order to reduce the possibility of false positive effects due to polymorphs in the probe sequence.

For *trans*-analysis, we first corrected the gene expression levels for the *cis*-effects to increase statistical power to detect *trans*-eQTL effects. The analyses was conducted on those SNPs present in the National Human Genome Research Institute GWAS catalog(11) (http://www.genome.gov/gwastudies, accessed 24/03/2015) and reported to be associated with complex traits or disease with a *P*-value of at most $5x10^{-8}$, referred to as GWAS SNPs in the text. Additionally, based on the National Human Genome Research Institute GWAS catalog we manually composed a list of GWAS SNPs associated with immune response.

We used the Spearman correlation coefficient (Spearman's rho) to detect associations between the coding allele of the SNP (directly genotyped or imputed allele dosages) and the variations in gene expression levels (residual gene expression levels obtained after corrections described before).

To control for multiple testing, we applied more conservative probe-level false discovery rate (FDR) procedure to account for the number of SNPs tested per probe (see the rationale for this approach in "Correction for multiple testing" block in the Supplementary Note by Westra *et al.*(10)). In short, we first generated a null distribution by 10 times performing eQTL mapping using permuted gene expression data with shuffled sample labels. Then, for both the real and permuted *P*-value distributions, we only used the most significant SNP per probe to determine the FDR at 0.05. We consider 10 permutations to be sufficient as it has been shown in Westra *et al.*(10) that FDR threshold estimate was quite stable after 5 permutations and adding additional permutations did not greatly change the significance

threshold. We assessed the significance thresholds for the *cis*- and *trans*-eQTL analysis separately.

References

1. Howie BN, Donnelly P, Marchini J. A flexible and accurate genotype imputation method for the next generation of genome-wide association studies. PLoS Genet. 2009 Jun;5(6):e1000529.

2. Tserel L, Kolde R, Limbach M, Tretyakov K, Kasela S, Kisand K, et al. Age-related profiling of DNA methylation in CD8+ T cells reveals changes in immune response and transcriptional regulator genes. Sci Rep. 2015 Jan;5:13107.

3. Trapnell C, Pachter L, Salzberg SL. TopHat: discovering splice junctions with RNA-Seq. Bioinformatics. 2009 May 1;25(9):1105–11.

4. R Core Team. R: A Language and Environment for Statistical Computing. Vienna, Austria: R Foundation for Statistical Computing; 2015.

5. Du P, Kibbe WA, Lin SM. lumi: a pipeline for processing Illumina microarray. Bioinformatics. 2008 Jul 1;24(13):1547–8.

6. Kauffmann A, Gentleman R, Huber W. arrayQualityMetrics--a bioconductor package for quality assessment of microarray data. Bioinformatics. 2009 Feb 1;25(3):415–6.

7. Westra H-J, Jansen RC, Fehrmann RSN, te Meerman GJ, van Heel D, Wijmenga C, et al. MixupMapper: correcting sample mix-ups in genome-wide datasets increases power to detect small genetic effects. Bioinformatics. 2011 Aug 1;27(15):2104–11.

8. Fairfax BP, Makino S, Radhakrishnan J, Plant K, Leslie S, Dilthey A, et al. Genetics of gene expression in primary immune cells identifies cell type-specific master regulators and roles of HLA alleles. Nat Genet. 2012 May;44(5):502–10.

9. Purcell S, Neale B, Todd-Brown K, Thomas L, Ferreira MAR, Bender D, et al. PLINK: a tool set for whole-genome association and population-based linkage analyses. Am J Hum Genet. 2007 Sep;81(3):559–75.

10. Westra H-J, Peters MJ, Esko T, Yaghootkar H, Schurmann C, Kettunen J, et al. Systematic identification of trans eQTLs as putative drivers of known disease associations. Nat Genet. 2013 Sep 8;45(10):1238–43.

11. Welter D, MacArthur J, Morales J, Burdett T, Hall P, Junkins H, et al. The NHGRI GWAS Catalog, a curated resource of SNP-trait associations. Nucleic Acids Res. 2014 Jan;42(Database issue):D1001–6.
